# AAV‐mediated gene therapy restores natural fertility and improves physical function in the *Lhcgr*‐deficient mouse model of Leydig cell failure

**DOI:** 10.1111/cpr.13680

**Published:** 2024-05-30

**Authors:** Suyuan Zhang, Bin Yang, Xiaoting Shen, Hong Chen, Fulin Wang, Zhipeng Tan, Wangsheng Ou, Cuifeng Yang, Congyuan Liu, Hao Peng, Peng Luo, Limei Peng, Zhenmin Lei, Sunxing Yan, Tao Wang, Qiong Ke, Chunhua Deng, Andy Peng Xiang, Kai Xia

**Affiliations:** ^1^ Center for Stem Cell Biology and Tissue Engineering, Key Laboratory for Stem Cells and Tissue Engineering, Ministry of Education, Sun Yat‐sen University Guangzhou Guangdong China; ^2^ National‐Local Joint Engineering Research Center for Stem Cells and Regenerative Medicine, Zhongshan School of Medicine, Sun Yat‐sen University Guangzhou Guangdong China; ^3^ Reproductive Medicine Center, The First Affiliated Hospital, Sun Yat‐sen University, The Key Laboratory for Reproductive Medicine of Guangdong Province Guangzhou Guangdong China; ^4^ Center for Stem Cells Translational Medicine, Shenzhen Qianhai Shekou Free Trade Zone Hospital Shenzhen Guangdong China; ^5^ Brain Cognition and Brain Disease Institute, Shenzhen Institutes of Advanced Technology, Chinese Academy of Sciences Shenzhen Guangdong China; ^6^ Department of Urology and Andrology The First Affiliated Hospital, Sun Yat‐sen University Guangzhou Guangdong China; ^7^ State Key Laboratory of Ophthalmology, Zhong Shan Ophthalmic Center, Sun Yat‐sen University Guangzhou Guangdong China; ^8^ Department of OB/GYN and Women's Health University of Louisville School of Medicine Louisville Kentucky USA; ^9^ Guangzhou Cellgenes Biotechnology Co., Ltd. Guangzhou Guangdong China

## Abstract

Leydig cell failure (LCF) caused by gene mutations leads to testosterone deficiency, infertility and reduced physical function. Adeno‐associated virus serotype 8 (AAV8)‐mediated gene therapy shows potential in treating LCF in the Lhcgr‐deficient (*Lhcgr* ^−/−^) mouse model. However, the gene‐treated mice still cannot naturally sire offspring, indicating the modestly restored testosterone and spermatogenesis in AAV8‐treated mice remain insufficient to support natural fertility. Recognizing this, we propose that enhancing gene delivery could yield superior results. Here, we screened a panel of AAV serotypes through in vivo transduction of mouse testes and identified AAVDJ as an impressively potent vector for testicular cells. Intratesticular injection of AAVDJ achieved markedly efficient transduction of Leydig cell progenitors, marking a considerable advance over conventional AAV8 vectors. AAVDJ‐Lhcgr gene therapy was well tolerated and resulted in significant recovery of testosterone production, substantial improvement in sexual development, and remarkable restoration of spermatogenesis in *Lhcgr* ^−/−^ mice. Notably, this therapy restored fertility in *Lhcgr* ^−/−^ mice through natural mating, enabling the birth of second‐generation. Additionally, this treatment led to remarkable improvements in adipose, muscle, and bone function in *Lhcgr* ^−/−^ mice. Collectively, our findings underscore AAVDJ‐mediated gene therapy as a promising strategy for LCF and suggest its broader potential in addressing various reproductive disorders.

## Graphical abstract

This study conducted AAV vectors scanning and pinpointed AAVDJ as highly efficient for targeting Leydig cell progenitors. Intratesticular injection of AAVDJ‐Lhcgr in the Leydig cell failure mouse model (*Lhcgr* ^−/−^) exhibited excellent tolerance, recovered testosterone production, resumed sexual development, rescued spermatogenesis, restored natural fertility, produced fertile offspring and improved physical function.

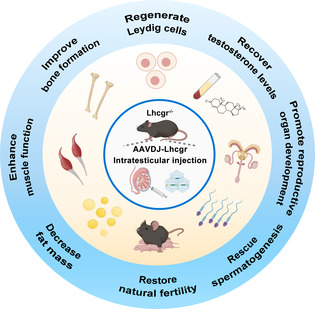

## 1 INTRODUCTION

Leydig cells (LCs) located in the testes are known to be responsible for over 95% of testosterone synthesis and secretion in men.^1^, ^2^ Thus, these cells are indispensable for the development and maintenance of the masculine phenotype, endocrine homeostasis, and reproductive function.^3^, ^4^ However, detrimental mutations in pivotal genes linked to testosterone synthesis can lead to reduced testosterone levels, which ultimately results in Leydig cell failure (LCF).^5^, ^6^ LCF typically manifests as a range of clinical symptoms, including micropenis, cryptorchidism, hypospadias, impaired spermatogenesis and infertility in affected individuals.^5^, ^7^ While testosterone replacement therapy (TRT) can elevate serum testosterone levels, it comes with the significant drawback of inhibiting the hypothalamic‐pituitary‐gonadal (HPG) axis through a negative feedback mechanism.^8^ As a consequence, TRT further hinders spermatogenesis and cannot address the issue of male infertility.^4^, ^9^ Given these challenges, there exists a pressing urgency to explore novel treatment modalities for LCF that can effectively provide viable solutions for male infertility.

A growing body of evidence highlights gene therapy as the most promising approach for treating genetic mutation‐related diseases.^10^ Among the various gene delivery vectors, the adeno‐associated virus (AAV) has gained widespread popularity due to its strong targeting capability, low immunogenicity and non‐integration into the host genome.^11^ Recently, in a pioneering proof‐of‐concept experiment using luteinizing hormone/choriogonadotropin receptor‐deficient (*Lhcgr* ^−/−^) LCF mice model, we found that AAV8‐mediated gene therapy targeted Leydig cell progenitors, increased testosterone levels, facilitated reproductive system development and restored spermatogenesis in *Lhcgr* ^−/−^ LCF mice.^12^ However, a limitation was evident as the serum testosterone levels of the treated *Lhcgr* ^−/−^ mice reached merely around 20% of those observed in wild‐type (WT) mice, and sperm counts in these treated mice were only about 50% of those in their WT counterparts. Collectively, these modest therapeutic effects, albeit promising, were proved inadequate to ensure natural fertility.^12^ To overcome these limitations, we propose a thorough examination of AAV serotypes, particularly emphasizing those that exhibit enhanced efficacy in targeting Leydig cell progenitors and amplifying gene expression.

Besides, reduced testosterone levels in LCF patients can also cause a constellation of related symptoms, including obesity,^13^ muscle atrophy^14^ and osteoporosis.^15^ These clinical manifestations not only impose considerable psychological stress but also exact a significant economic toll on affected patients.^16^ Previous studies have demonstrated that *Lhcgr* ^−/−^ male mice develop obesity and osteoporosis,^17^ closely mirroring the phenotype seen in human LCF. Given these insights, exploring the potential of AAV‐mediated gene therapy to improve the functions of adipose tissue, muscle, and bone in LCF mice emerges as a significant avenue that has yet to be explored.

In the present study, we conducted an extensive screening of AAV vectors to identify those exhibiting enhanced targeting efficiency for Leydig cell progenitors and assessed the impact of AAV‐mediated gene therapy on testosterone levels, sexual development, and spermatogenesis in LCF mice. Building upon this, our investigation aimed to determine whether LCF mice could achieve fertility and produce offspring through natural mating following gene therapy. Additionally, we explored the functional improvements in adipose tissue, muscle, and bone in LCF mice after gene therapy.

## 2 MATERIALS AND METHODS

### 2.1 Animals

The male *Lhcgr* ^+/+^ and *Lhcgr* ^−/−^ mice used in this study were derived from a breeding colony of *Lhcgr* ^+/−^ C57BL/6 mice, generously provided by Z. L.^18^ Genotyping of the mice was performed using PCR on DNA extracted from tail samples, as previously described.^12^ *Lhcgr* ^−/−^ mice were randomly assigned to experimental groups. All animals were housed in the Sun Yat‐sen University Animal Center under specific conditions of constant temperature (24 ± 1°C), relative humidity (50%–60%), and a 12 h light/12 h dark cycle. They were provided ad libitum access to food and water throughout the study. The animal experiments were conducted following ethical guidelines and were approved by the First Affiliated Hospital of Sun Yat‐sen University (Approval No. 2020‐003). Strict adherence to animal welfare and ethical considerations was ensured throughout the study.

### 2.2 Gene delivery in animal models

AAVDJ and AAV8 vectors, encompassing the full‐length complementary DNA (cDNA) of mouse Lhcgr, were procured from Vigene Bioscience (Shandong, China), along with vectors encoding mCherry under the regulation of the CAG promoter. For in vivo administration, either phosphate‐buffered saline (PBS) or AAV particles were injected into the interstitial space of individual testes following an established protocol.^12^ In brief, *Lhcgr* ^−/−^ mice were anaesthetized with Avertin (250 mg/kg) via intraperitoneal (i.p.) injection. The surgical site was subsequently sterilized using ethanol and povidone‐iodine applied topically. Under aseptic conditions, a single incision was made in the ventral skin and body wall anterior to the genitals using surgical scissors. The epididymal fat was then carefully manipulated to extricate the testes without damaging the associated blood vessels. Each testis was then immobilized with fine forceps, and a standardized dose of 8 μL/testis was administered through a 33‐gauge needle syringe (Hamilton, Switzerland). The procedure was finalized with the suturing of the incision.

### 2.3 RNA isolation, cDNA synthesis and qRT‐PCR

Total RNA was isolated from mouse testes using an RNA Quick Purification Kit (YiSan Biotech, Shanghai, China). Briefly, the purity and concentration of the total RNA were assessed using a NanoDrop 8000 spectrophotometer (Thermo Fisher Scientific, Wilmington, DE, USA), and 1 μg of total RNA was reverse transcribed. cDNA was synthesized following the manufacturer's instructions using the NovoScript® 1st Strand cDNA Synthesis Kit (Novoprotein, Shanghai, China). Quantitative real‐time PCR (qRT‐PCR) was performed to quantify target mRNA levels. The reactions were carried out with the LightCycler® 480 SYBR Green I Master (Roche, Indianapolis, IN, USA) on a LightCycler 480 Detection System (Roche) following the manufacturer's protocols. To validate the primers, a melting curve analysis was performed to ensure the presence of a single peak and exclude the possibility of non‐specific products or primer dimer formation. All samples were analysed in triplicate, and target mRNA levels were calculated using the ΔCt method, with β‐actin serving as the internal control. The primer sequences used for qRT‐PCR are provided in Table S1.

### 2.4 Immunofluorescence staining

Immunofluorescence staining was performed following previously reported protocols from our group.^19^ Testis samples were fixed in 4% paraformaldehyde (PFA, Phygene, Fuzhou, China) for 4 h at 4°C and subsequently dehydrated using 30% sucrose solution (Sangon Biotech, Shanghai, China) for 24 h at 4°C. The tissues were then embedded in Tissue‐Tek O.C.T. Compound (Sakura Finetek, Torrance, CA, USA), frozen, and cryosectioned into 10 μm thick slices using a frozen slicer (Leica CM1950). For heat‐induced antigen retrieval, sodium citrate antigen retrieval solution (Beyotime, Shanghai, China) was used in a steamer for 15 min. The sections were permeabilized with 0.2% Triton X‐100 (Sigma, St. Louis, MO, USA) for 15 min and then blocked with 10% goat serum (Boster, Wuhan, Hubei, China) and 2% bovine serum albumin (BSA, Sigma) in PBS for 45 min at room temperature. The sections were incubated with primary antibodies, including Rabbit anti‐PDGFRα (1:200; Abcam, no. ab203491), Mouse anti‐NESTIN (1:200; Millipore, no. MAB353), Chicken anti‐mCherry (1:400; Abcam, no. ab205402), Rabbit anti‐α‐SMA (1:400; Abcam, no. 5464), Rabbit anti‐DDX4 (1:400; Cell Signaling Technology, no. 8761s), Rabbit anti‐CYP17A1 (1:400; Cell Signaling Technology, no. 94004s), Rabbit anti‐AIF1 (1:400; GeneTex, no. GTX100042), Mouse anti‐LHCGR (1:200; Novus, no. NBP2‐54479), Mouse anti‐TNP2 (1:200; Santa Cruz, no. SC‐393843), Rat Anti‐Mouse CD4 (1:50; BD, no. 557667), Rat Anti‐Mouse CD8a (1:50; BD, no. 557668), Rabbit anti‐MPO (1: 200, Abcam, no. ab208670) overnight at 4°C. After washing with PBS, the sections were incubated with the appropriate secondary antibodies in the dark at room temperature for 1 h (Goat Anti‐rabbit IgG AF488, 1:1000, no. A11037; Goat Anti‐rabbit IgG AF647, 1:1000, no. A32733; Goat Anti‐mouse IgG AF488, 1:1000, no. A10680; Goat Anti‐mouse IgG AF647, 1:1000, no. A32728; Goat Anti‐chicken IgG AF555, 1:1000, no. A32932; Thermo Fisher Scientific) and co‐stained with DAPI (Gibco) for 5 min. Images were captured using an LSM800 confocal microscope (Zeiss, Jena, Germany) or a Leica DMi8 microscope (Leica, Wetzlar, Germany).

### 2.5 Labelling Leydig cell progenitors with Click‐iT EdU in vivo

The proliferation and differentiation of Leydig cell progenitors after AAVDJ injection in *Lhcgr* ^*−/−*^ mice were evaluated with a Click‐iT®EdU Cell Fluor Cell Proliferation Assay Kit (Invitrogen, USA) according to the manufacturer's instructions. To characterize the proliferative response of Leydig cell progenitors after AAV injection (Day 0), the *Lhcgr* ^−/−^ mice received daily injections of EdU (50 mg/kg) for five consecutive days (from Day 3 to Day 7). Subsequently, the testicular samples were collected at Day 8 and subjected to immunostaining analysis for EdU (proliferative cells from Day 3 to Day 7), mCherry (AAV targeted cells) and PDGFRα (Leydig cell progenitors). To detect the differentiation of Leydig cell progenitors into LCs after AAV injection, the testicular samples were collected at Day 12 and subjected to immunostaining analysis for EdU (proliferative cells from Day 3 to Day 7), mCherry (AAV targeted cells) and CYP17A1 (LCs).

### 2.6 Haematoxylin and eosin staining

Testis and epididymis samples were collected and fixed in Bouin's solution (Sigma) overnight. After fixation, the samples were dehydrated in 75% ethanol, embedded in paraffin and sectioned into 4 μm thick slices. The paraffin‐embedded sections were deparaffinized using xylene and then gradually rehydrated with a series of ethanol concentrations. For histological analysis, the prepared sections were stained with haematoxylin and eosin (H&E). The stained sections were examined using an AxioScan.Z1 microscope (Zeiss) or a Leica DMi8 microscope (Leica).

### 2.7 Sex hormone assays

Sex hormone concentrations were measured as previously reported by our group.^12^ Blood and testes were collected at specific timepoints. The blood and grinded testes were centrifuged to separate the supernatant, which was then stored at −80°C for further analysis. Testosterone levels in both serum and testes were determined using a chemiluminescent immunoassay (CLIA) provided by KingMed Diagnostics Group Co., Ltd. (Guangzhou, China). The coefficient of variation (CV) for intra‐assay precision ranged from 1.9% to 5.1%, and that for inter‐assay precision ranged from 2.5% to 5.2%. The minimum detectable concentration of testosterone is 0.01 ng/mL. The concentrations of insulin‐like peptide 3 (Insl3), luteinizing hormone (LH) and follicle‐stimulating hormone (FSH) were measured using specific ELISA assay kits. Mouse Insl3 ELISA Kit (Phoenix, no. EK‐035‐43), mouse LH ELISA Kit (Cloud‐Clone Corp., no. BWN‐Uscnk‐CEA441Mu) and mouse FSH ELISA Kit (Cloud‐Clone Corp., no. BWN‐Uscnk‐CEA830Mu) were used following the manufacturer's protocols. The optical density (OD) values were recorded at 450 ± 10 nm and measured by a microplate reader (Sunrise, TECAN). The absolute concentrations were calculated according to the standard curve.

### 2.8 Computer‐aided semen analysis

Semen samples underwent rigorous quantitative analysis in accordance with well‐defined protocols.^20^ For each mouse, one cauda epididymis was surgically excised and incised utilizing micro‐scissors and then incubated in 0.5 mL DMEM/F12 containing 0.5% BSA (Sigma) for 15 min at 37°C, to facilitate the liberation of spermatozoa from the epididymis. After incubation, the tissue was promptly discarded, and the rest of the mixture was suitably diluted for subsequent analysis using a Hamilton Thorne Ceros II system (Hamilton Thorne, Massachusetts, USA). For the analytical procedure, no fewer than six fields of each sample were arbitrarily assessed to ascertain both the concentrations of sperm and the proportion of sperm exhibiting motility and progressive motility.

### 2.9 Assessment of reproductive function

To assess fertility, male *Lhcgr* ^−/−^ mice that received AAVDJ‐Lhcgr were co‐housed with fertility‐proven female *Lhcgr* ^+/+^ mice in a cage, and the first‐generation offspring (F1) were recorded after 6 weeks. Subsequently, when F1 mice reached sexual maturity, each of them was individually housed with one *Lhcgr* ^+/+^ mouse of the opposite sex to produce the second‐generation offspring (F2). The numbers of litters and pups per litter were recorded over the course of the next 4 months.

### 2.10 Analysis of AAVDJ‐Lhcgr integration in offspring

The offspring were subjected to screening for the presence of the transgene AAVDJ construct through PCR analysis as previously described.^21^ Genomic DNA was extracted from the tails of the offspring using the Trelief® Mouse Direct PCR Kit (Tsingke Biotechnology Co., Ltd.) following the manufacturer's instructions. PCR was performed using specific primers for the CAG promoter and the inserted Lhcgr gene, using a Bio‐Rad T100 thermal cycler according to the manufacturer's protocol. The primers used for PCR were listed in Table S1. The PCR products were subjected to separation by electrophoresis within a 1.3% agarose gel infused with ethidium bromide. Following this procedure, the separated bands were visualized employing ultraviolet transillumination, and images were captured using Tanon 2000B (Tanon, Shanghai, China).

### 2.11 Analysis of muscle and adipose tissue

The tibialis anterior muscle and epididymal adipose tissue were isolated, and their masses were measured and documented. The tissues were then fixed with 4% PFA overnight at 4°C. After fixation, the tissues were embedded in paraffin and sliced to obtain maximum cross‐sections with a thickness of 5 μm. To analyse the areas of myofibers and adipose tissue per unit of field, the paraffin sections were stained with H&E. ImageJ software (V1.8.0.112) was used for the analysis, and the areas of myofibers and adipose tissue were quantified in square micrometres (μm^2^) per unit of field.

### 2.12 Grip strength test

To assess the grip strength of the mice, a grip strength meter apparatus (Chatillon) was utilized following established protocols.^22^ Before the testing, the strength meter was positioned horizontally and set to the maximal grip strength mode. For measurement of forelimb grip strength, the mouse was gently lowered over the top of the grid so that only its front paws could grip the grid. The mouse's trunk was kept horizontal and parallel to the grid, and then it was pulled backward steadily. The maximal grip strength value of the animal was recorded when it released the grid. Each mouse underwent three repeated trials followed by a 10 min rest as a round of testing. A total of five rounds were executed, and the mean of the top three highest values among the resultant 15 grip strength measurements was calculated, representing the maximal grip strength for each individual mouse.

### 2.13 Endurance test

To assess muscle endurance, a hanging test was conducted following established procedures.^22^ Each mouse was lifted by the tail and placed on a square grid with a 1 cm mesh size. The grid was then inverted to a height of 40 cm over a soft pad, and the mouse was allowed to hang by all of its paws. The duration that the mouse was able to hang was recorded during a 600 s test period, which was the maximal test duration. For each mouse, three repeated trials were conducted with a 10 min rest between each trial. The hanging time for each trial was recorded, and the average of the three recorded values was used as the hanging time for each mouse.

### 2.14 Treadmill test

Treadmill performance was assessed using a Rotamax Treadmill (Columbus Instruments) following established protocols.^23^ To acclimate the mice to the treadmill, each mouse was placed on the treadmill running at a low speed of 10 m/min for 15 min, repeated twice a day for three consecutive days. On the test day, mice were warmed up at a speed of 5 m/min for 2 min before the experimental running. The test session commenced at a speed of 10 m/min for 5 min, and the speed was gradually increased by 2 m/min every 2 min to the maximum speed of 46 m/min until the mice were exhausted. Mice were considered exhausted when they were not capable of returning to the treadmill despite the electrical and mechanical stimulation. The distance (m) was recorded to calculate work throughout the test according to the following formula: work (J) = mass (g) × g (9.81 m/s^2^) × distance (m) × sin (15°).

### 2.15 Micro‐CT analysis

Micro‐CT analysis was performed following previously established protocols.^24^ Femora were dissected from the mice and fixed in 4% PFA for 24 h. The femora were then analysed using a Micro‐CT system (Siemens). The software Inveon Research Workplace (Siemens) was utilized to measure the number, thickness, and spacing of the trabecular bone per given layer. Two‐dimensional images of the femora in the horizontal and coronal planes, as well as three‐dimensional images of bone trabecula, were then documented.

### 2.16 Statistical analysis

All data were subjected to statistical analysis using IBM SPSS Statistics version 25.0 software (IBM SPSS Statistics, Armonk, NY, USA) and the results were visualized using GraphPad Prism 9 software (GraphPad Software, La Jolla, CA, USA). Statistical comparisons between two groups were conducted using the unpaired *t*‐test. For comparisons among multiple groups, one‐way analysis of variance (ANOVA) was employed. Differences were considered significant when *p* < 0.05 (*p < 0.05; **p < 0.01; ***p < 0.001), ns = not significant.

## 3 RESULTS

### 3.1 Intratesticular injection of AAVDJ efficiently targets Leydig cell progenitors

We screened 11 types of AAVs with different capsids (AAV1, 2, 5, 6, 7, 7m8, 8, 9, rh10, anc80 and DJ), all of which encode mCherry driven by the CAG promoter. These AAV particles were subsequently microinjected into the interstitium of the testes at varying doses of 8 × 10^8^, 8 × 10^9^, and 8 × 10^10^ genome copies per testis (gc/testis), respectively. Fluorescence detection of the testes was carried out 7 days after vector exposure, and the results demonstrated relatively robust signals in the testes that received interstitial injections of AAV1, AAV6, AAV8 and AAVDJ (Figure S1). Notably, mCherry expression exhibited superior brightness in testes exposed to AAVDJ, with detectable signals even at the lowest dose (8 × 10^8^ gc/testis) (Figure S1). These findings indicate that AAVDJ drives the most potent gene expression within the testes.

To assess the infection efficiency and specificity of AAVDJ in testicular cells, we injected AAVDJ carrying the CAG‐mCherry reporter vector at varying doses (8 × 10^8^, 8 × 10^9^ and 8 × 10^10^ gc/testis) into the testes of 8‐week‐old *Lhcgr* ^−/−^ mice. In parallel, we utilized AAV8‐mCherry vector (8 × 10^10^ gc/testis) as a control, as previously reported.^12^ 7 days after AAV vector injection, we collected the testes and conducted immunofluorescence analysis. Through co‐expression analysis of mCherry and Leydig cell progenitors markers, platelet‐derived growth factor receptor alpha (PDGFRα) and Nestin, we observed a dose‐dependent increase in AAVDJ infection efficiency in Leydig cell progenitors (Figure S2A,B). At doses of 8 × 10^9^ and 8 × 10^10^ gc/testis, AAVDJ effectively transfected Leydig cell progenitors, surpassing the infection rate observed in the high‐dose AAV8 (8 × 10^10^ gc/testis) injection group (Figure S2C,D). These findings indicate that AAVDJ exhibits higher infection efficiency in Leydig cell progenitors compared to AAV8.

To assess the transduction potential of AAVDJ in germ cells, we conducted immunostaining analysis of testicular tissue injected with the highest dose of AAVDJ‐mCherry (8 × 10^10^ gc/testis). Seven days after injection, our immunostaining analysis demonstrated that mCherry expression did not co‐localize with the germ cell marker DEAD‐box helicase 4 (DDX4), indicating the absence of AAVDJ infection in this specific cell type (Figure S2E). Additionally, minimal mCherry expression was observed in cells expressing the macrophage marker allograft inflammatory factor 1 (AIF1) and the peritubular myoid cell marker alpha‐smooth muscle actin (a‐SMA), implying a low likelihood of AAVDJ transduction in these cell types (Figure S2F,G). Moreover, we thoroughly examined the possibility of off‐target AAVDJ transduction in other tissues following testicular injection. Immunofluorescence analysis revealed that mCherry expression was confined to the testis and not detected in the liver, heart, muscle, kidney, or colon (Figure S3). These observations suggest that intratesticularly injected AAVDJ exhibits favourable tropism to testis in comparison to other organs. Furthermore, we investigated the presence of lymphocytes, macrophages and neutrophils 7 days after AAVDJ‐mCherry injection. Our analysis revealed no significant difference in the number of inflammatory cells between the groups with and without injection (Figure S4), implying that intratesticular injection of AAVDJ is safe and well tolerated.

### 3.2 AAVDJ‐Lhcgr restores Lhcgr expression and recovers testosterone levels in *Lhcgr* ^−/−^ mice

**FIGURE 1:**
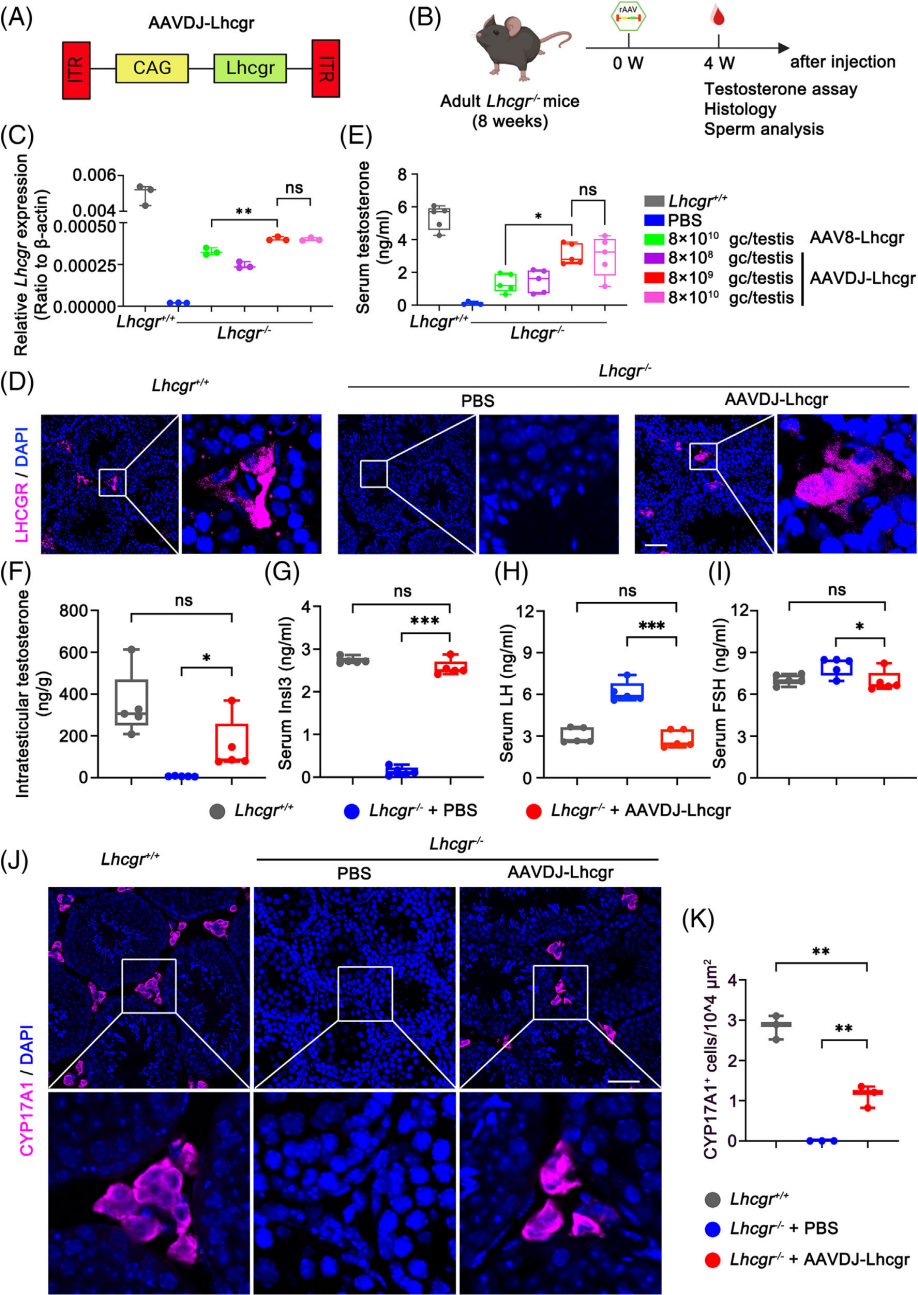
AAVDJ‐Lhcgr restores Lhcgr expression and testosterone levels in *Lhcgr* ^−/−^ mice. (A) Schematic of the AAV vector used in the study. (B) Experimental overview of the in vivo studies. (C) qRT‐PCR was used to quantify Lhcgr mRNA transcripts in testicular tissues from *Lhcgr* ^+/+^ mice and *Lhcgr* ^−/−^ mice injected with PBS or increasing doses of AAVDJ‐Lhcgr (8 × 10^8^, 8 × 10^9^ and 8 × 10^10^ gc/testis) or AAV8‐Lhcgr (8 × 10^10^ gc/testis) 4 weeks after treatment (*n* = 3). β‐actin was used for normalization. (D) Representative images of LHCGR in the testes of *Lhcgr* ^+/+^ mice, *Lhcgr* ^−/−^ mice injected with PBS or AAVDJ‐Lhcgr (8 × 10^9^ gc/testis) 4 weeks after treatment (*n* = 4). The nuclei were counterstained with DAPI. Scale bar: 50 μm. (E) The concentrations of serum testosterone were analysed in *Lhcgr* ^+/+^ mice and *Lhcgr* ^−/−^ mice injected with PBS or increasing doses of AAVDJ‐Lhcgr (8 × 10^8^, 8 × 10^9^ and 8 × 10^10^ gc/testis) or AAV8‐Lhcgr (8 × 10^10^ gc/testis) 4 weeks after treatment (*n* = 5). (F) The concentrations of intratesticular testosterone were detected in *Lhcgr* ^+/+^ mice and *Lhcgr* ^−/−^ mice injected with PBS or AAVDJ‐Lhcgr (8 × 10^9^ gc/testis) 4 weeks after treatment (*n* = 5). (G–I) The concentrations of serum Insl3 (G), LH (H) and FSH (I) were analysed in *Lhcgr* ^+/+^ mice and *Lhcgr* ^−/−^ mice injected with PBS or AAVDJ‐Lhcgr (8 × 10^9^ gc/testis) 4 weeks after treatment (*n* = 5). (J) CYP17A1 was evaluated by immunostaining of testes from *Lhcgr* ^+/+^ mice, *Lhcgr* ^−/−^ mice injected with PBS or AAVDJ‐Lhcgr (8 × 10^9^ gc/testis) 4 weeks after treatment (*n* = 3). The nuclei were counterstained with DAPI. Scale bar: 50 μm. (K) CYP17A1^+^ cells were quantified in the different groups. Data are represented by boxplots, and whiskers show the minimum to maximum values. *p < 0.05; **p < 0.01; ***p < 0.001. ns, not significant.

To explore the relative efficacy of AAVDJ and AAV8 in restoring Lhcgr expression and testosterone levels in *Lhcgr* ^−/−^ mice, we developed an AAV vector containing the mouse Lhcgr sequence driven by the CAG promoter (Figure 1A). To assess the therapeutic effects of these vectors, we organized 8‐week‐old *Lhcgr* ^−/−^ mice into distinct groups, including a PBS injection group, an AAV8‐Lhcgr injection group (at a dose of 8 × 10^10^ gc/testis), and AAVDJ‐Lhcgr injection groups (at doses of 8 × 10^8^, 8 × 10^9^ and 8 × 10^10^ gc/testis, respectively) (Figure 1B). Additionally, we utilized age‐matched *Lhcgr* ^+/+^ mice that underwent a sham operation as controls.

To assess the efficacy of gene delivery, we examined Lhcgr mRNA and protein expression levels in testicular tissue 4 weeks post‐treatment. qRT‐PCR analysis of testicular tissue revealed a dose‐dependent increase in Lhcgr transcripts in the testes of AAVDJ‐Lhcgr‐treated *Lhcgr* ^−/−^ mice, while Lhcgr expression remained undetectable in *Lhcgr* ^−/−^ mice treated with PBS (Figure 1C). Notably, Lhcgr mRNA expression in the testes of AAVDJ‐Lhcgr‐treated *Lhcgr* ^−/−^ mice at a dose of 8 × 10^9^ gc/testis was significantly higher than that observed in the AAV8‐treated group at a dose of 8 × 10^10^ gc/testis (Figure 1C). Subsequently, immunofluorescence staining demonstrated obvious LHCGR protein expression in the testicular interstitium of the AAVDJ‐Lhcgr‐treated group (at a dose of 8 × 10^9^ gc/testis), while LHCGR protein expression was nearly undetectable in the testicular interstitium of the *Lhcgr* ^−/−^ mice treated with PBS (Figure 1D). Collectively, these results illustrate the ability of AAVDJ‐Lhcgr treatment to restore Lhcgr expression in the testes of *Lhcgr* ^−/−^ mice.

In addition, the serum testosterone concentration of *Lhcgr* ^−/−^ mice in the AAVDJ‐Lhcgr treatment group increased significantly and peaked at a dose of 8 × 10^9^ gc/testis, reaching 60% of the serum testosterone concentration of *Lhcgr* ^+/+^ mice (Figure 1E). This increase was notably higher than that observed in the AAV8‐Lhcgr treatment group (8 × 10^10^ gc/testis) (Figure 1E). In subsequent experiments, we selected 8 × 10^9^ gc/testis of AAVDJ‐Lhcgr as the gene therapy dose. The level of intratesticular testosterone, which is crucial for spermatogenesis, was also markedly elevated in the AAVDJ‐Lhcgr‐treated group (8 × 10^9^ gc/testis) compared to the PBS‐treated group 4 weeks post‐treatment (Figure 1F). Insl3, a peptide hormone secreted by mature LCs, serves as a marker for assessing LCs maturation.^25^ To evaluate the effect of gene therapy on LCs maturation, we measured Insl3 levels in the serum and observed a significant increase 4 weeks after AAVDJ‐Lhcgr injection (Figure 1G). Additionally, we assessed LH and FSH levels and found a significant decrease in LH and FSH levels compared to the PBS‐treated group 4 weeks after AAVDJ‐Lhcgr injection (Figure 1H,I). The testosterone production in AAV‐Lhcgr treated *Lhcgr* ^−/−^ mice was regulated by the HPG (Figure S5). Moreover, we analysed the expression level of the LCs marker CYP17A1 in the testis 4 weeks after AAVDJ‐Lhcgr injection using immunofluorescence staining. The results revealed a significantly higher number of CYP17A1^+^ LCs in the testicular interstitium of *Lhcgr* ^−/−^ mice treated with AAVDJ‐Lhcgr compared to those treated with PBS (Figure 1J,K). Besides, AAVDJ‐Lhcgr treatment promoted the proliferation and differentiation of Leydig cell progenitors as shown by the increase of EdU incorporation in mCherry^+^PDGFRα^+^ cells and mCherry^+^CYP17A^+^ cells in the testes of *Lhcgr* ^−/−^ mice (Figure S6). In summary, AAVDJ‐Lhcgr treatment successfully recovers the function of LCs and restores testosterone levels in *Lhcgr* ^−/−^ mice.

### 3.3 AAVDJ‐Lhcgr promotes reproductive organ development in *Lhcgr* ^−/−^ mice

**FIGURE 2:**
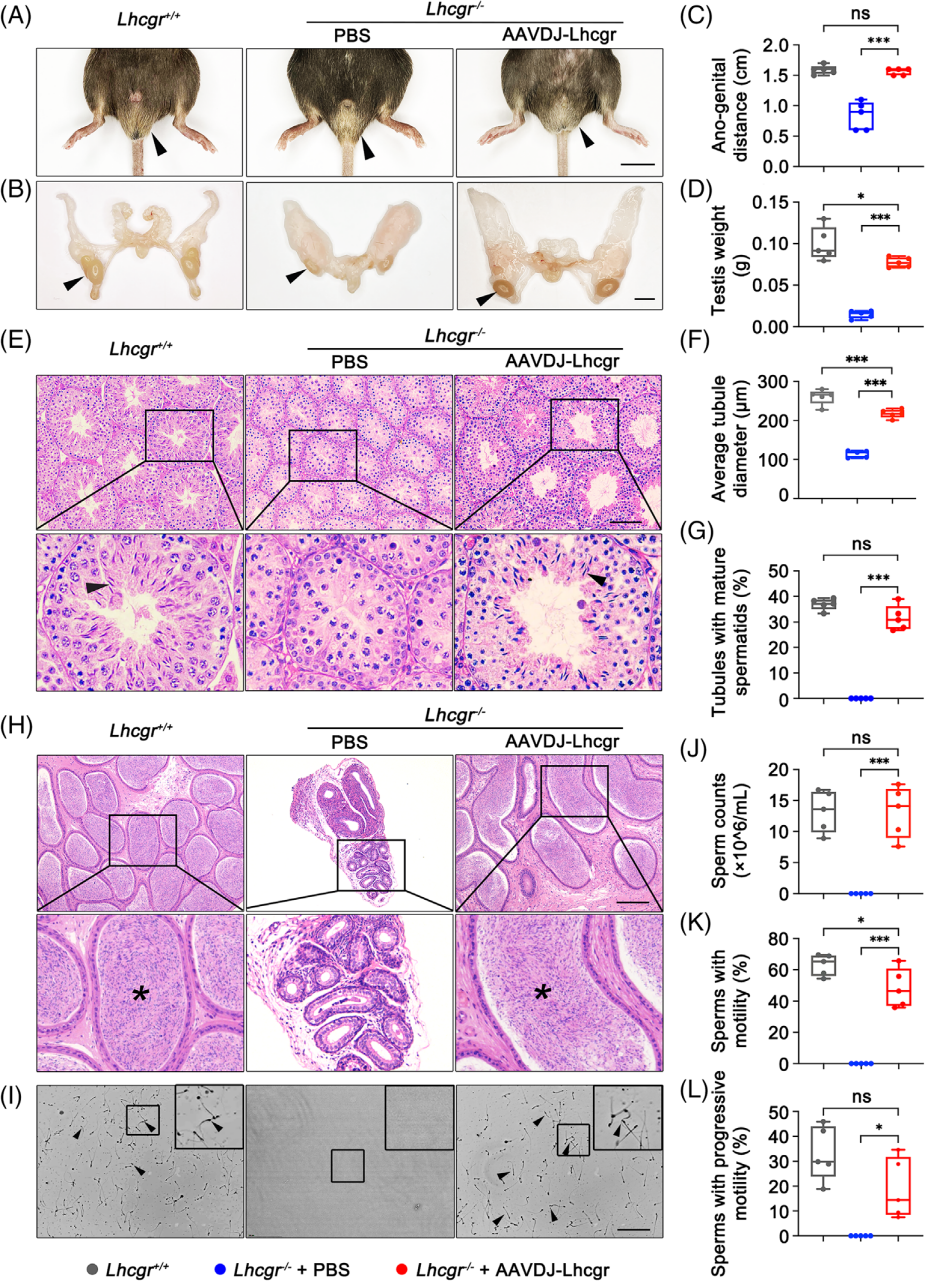
AAVDJ‐Lhcgr restarts sexual development and rescues spermatogenesis in *Lhcgr* ^−/−^ mice. (A and B) Representative photographs of the external (A) and internal genitalia (B) of *Lhcgr* ^+/+^ mice and *Lhcgr* ^−/−^ mice injected with PBS or AAVDJ‐Lhcgr (8 × 10^9^ gc/testis) 4 weeks after treatment (*n* = 5). Arrowheads indicate the testes. Scale bar: 0.5 cm. (C and D) Quantification of ano‐genital distance (C) and testis weight (D) of *Lhcgr* ^+/+^ mice and *Lhcgr* ^−/−^ mice injected with PBS or AAVDJ‐Lhcgr (8 × 10^9^ gc/testis) 4 weeks after treatment (*n* = 5). (E) Representative micrographs of testicular sections from *Lhcgr* ^+/+^ mice and *Lhcgr* ^−/−^ mice injected with PBS or AAVDJ‐Lhcgr (8 × 10^9^ gc/testis) 4 weeks after treatment (*n* = 5). Scale bar: 100 μm. Arrowheads indicate full spermatogenesis in testes. (F and G) The average tubular diameter (F) and the percentage of tubules with mature spermatids (G) were analysed in *Lhcgr* ^+/+^ mice and *Lhcgr* ^−/−^ mice injected with PBS or AAVDJ‐Lhcgr (8 × 10^9^ gc/testis) 4 weeks after treatment (*n* = 5). (H) Histological analysis of cauda epididymis collected from *Lhcgr* ^+/+^ mice and *Lhcgr* ^−/−^ mice injected with PBS or AAVDJ‐Lhcgr (8 × 10^9^ gc/testis) 4 weeks after treatment (*n* = 5). Stars indicate spermatozoa in the cauda epididymis. Scale bar: 100 μm. (I) Representative light micrographs of sperm obtained from the cauda epididymis of *Lhcgr* ^+/+^ mice and *Lhcgr* ^−/−^ mice injected with PBS or AAVDJ‐Lhcgr (8 × 10^9^ gc/testis) 4 weeks after treatment (*n* = 5). Scale bar: 100 μm. (J–L) The sperm counts (J), proportions of sperm with motility (K) and progressive motility (L) were analysed in *Lhcgr* ^+/+^ mice and *Lhcgr* ^−/−^ mice injected with PBS or AAVDJ‐Lhcgr (8 × 10^9^ gc/testis) 4 weeks after treatment (*n* = 5). Data are represented by boxplots, and whiskers show the minimum to maximum values. *p < 0.05; ***p < 0.001. ns, not significant.

Based on the observation of the positive effects of AAVDJ‐Lhcgr therapy on testosterone levels in *Lhcgr* ^−/−^ mice, we extended our investigation to evaluate the impact of gene therapy on the development of reproductive organs in these mice. Four weeks after AAVDJ‐Lhcgr treatment (8 × 10^9^ gc/testis), we noted significant improvements in various reproductive organ parameters. Specifically, the testes descended to the scrotum, and the genitalia of the mice displayed further development (Figure 2A,B). The ano‐genital distance in *Lhcgr* ^−/−^ mice of the AAVDJ‐Lhcgr treatment group was also significantly higher compared to the PBS‐treated group (Figure 2C), further supporting the promotion of masculinization by gene therapy. Moreover, AAVDJ‐Lhcgr treatment resulted in increased testis weight and penile length compared to PBS‐treated *Lhcgr* ^−/−^ mice (Figure 2D; Figure S7A). The hypoplastic epididymis of *Lhcgr* ^−/−^ mice exhibited significant enlargement after AAVDJ treatment, with its weight reaching the level observed in *Lhcgr* ^+/+^ mice (Figure S7B). Furthermore, the weight of the seminal vesicles in the AAVDJ‐Lhcgr group increased, while the seminal vesicles remained macroscopically undetectable in PBS‐treated *Lhcgr* ^−/−^ mice (Figure S7C). Additionally, the weight of the levator ani/bulbocavernosus (LA/BC) muscles in AAVDJ‐Lhcgr‐treated *Lhcgr* ^−/−^ mice was significantly higher than that in mice from the PBS‐treatment group (Figure S7D). Collectively, these results provide compelling evidence that AAVDJ‐Lhcgr gene therapy effectively restarts the development of reproductive organs in *Lhcgr* ^−/−^ mice.

### 3.4 AAVDJ‐Lhcgr rescues spermatogenesis in *Lhcgr* ^−/−^ mice

Given the successful restoration of testosterone levels and promotion of reproductive organ development in *Lhcgr* ^−/−^ mice following AAVDJ‐Lhcgr treatment, we subsequently determined whether AAVDJ‐Lhcgr therapy could rescue spermatogenesis in *Lhcgr* ^−/−^ mice. Histological analysis revealed that the diameter of seminiferous tubules in PBS‐treated *Lhcgr* ^−/−^ testes were significantly reduced, and spermatogenesis was arrested, with a lack of mature spermatozoa (Figure 2E–G). In contrast, the testes of the AAVDJ‐Lhcgr treatment group (8 × 10^9^ gc/testis) exhibited a significant increase in the diameter of seminiferous tubules, and spermatogenesis was evident, with the presence of mature spermatozoa in these testes (Figure 2E–G).

To further assess the impact of gene therapy on spermatogenesis, cauda epididymal samples were collected from the three groups of mice 4 weeks after treatment. Histological analysis demonstrated that *Lhcgr* ^−/−^ mice in the PBS‐treated group exhibited a significantly smaller lumen diameter of the cauda epididymis and a complete absence of sperm in the lumen when compared to *Lhcgr* ^+/+^ mice (Figure 2H). In contrast, *Lhcgr* ^−/−^ mice in the AAVDJ‐Lhcgr‐treated group displayed a significantly larger diameter of the cauda epididymal lumen, and the lumen contained an abundance of sperm (Figure 2H). To further quantitate spermatogenesis following AAVDJ‐Lhcgr treatment, we employed a computer‐aided semen analysis (CASA) system to examine the quantity and motility of sperm. The results revealed that after 4 weeks of treatment, the epididymal sperm count in the AAVDJ‐Lhcgr group exhibited a significant increase, reaching the same level as the sperm count observed in *Lhcgr* ^+/+^ mice (Figure 2I,J). However, no sperm were detected in the epididymis of PBS‐treated *Lhcgr* ^−/−^ mice (Figure 2I,J). Besides, the sperm of AAVDJ‐Lhcgr‐treated *Lhcgr* ^−/−^ mice exhibited considerable motility and progressive motility, almost reaching the level observed in *Lhcgr* ^+/+^ mice (Figure 2K,L). These results indicate that AAVDJ‐Lhcgr gene therapy effectively rescues spermatogenesis, resulting in substantial increases in sperm quantity and motility.

**FIGURE 3:**
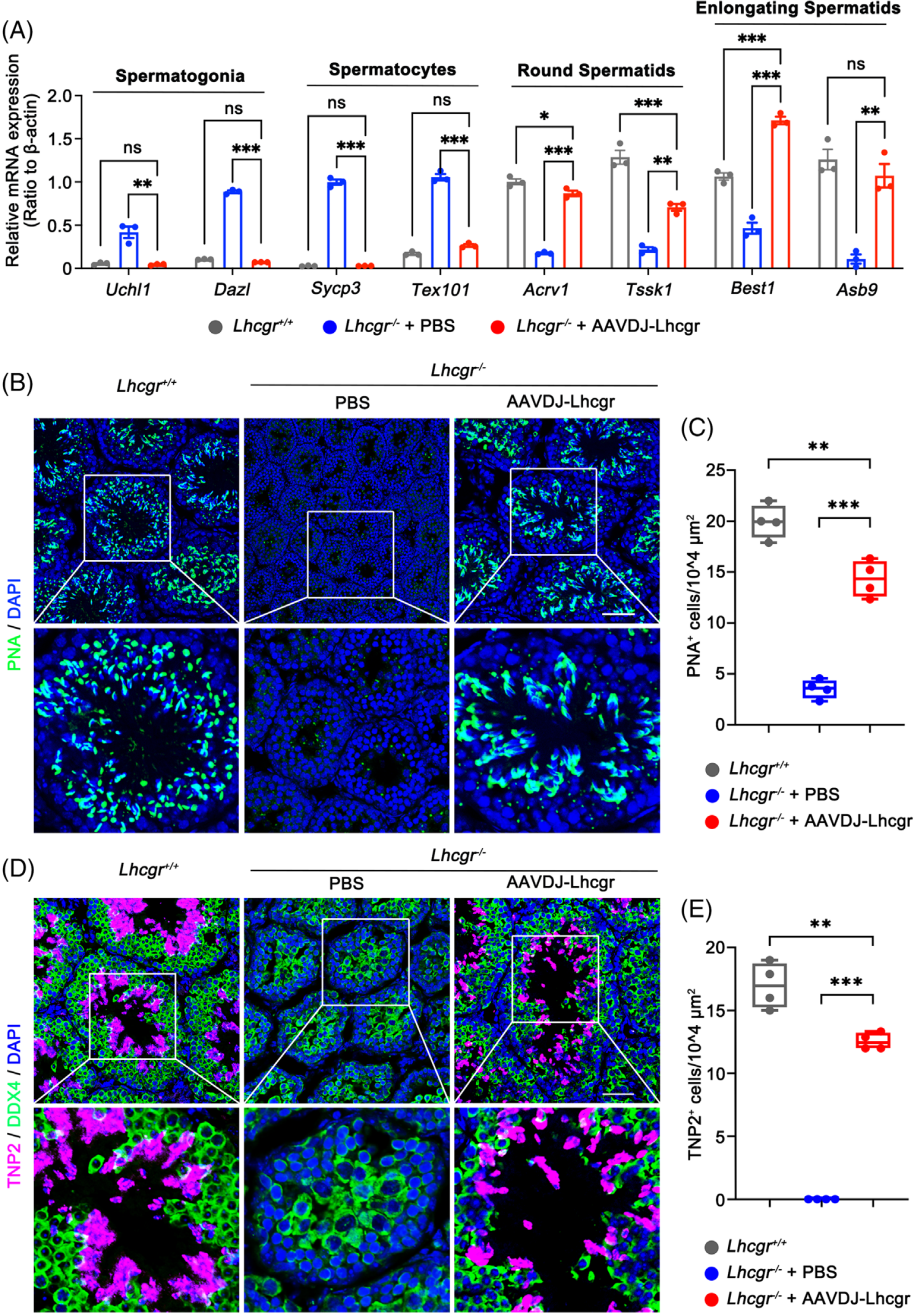
AAVDJ‐Lhcgr promotes the formation of round spermatids and elongating spermatids. (A) qRT‐PCR was performed in testicular samples collected from *Lhcgr* ^+/+^ mice and *Lhcgr* ^−/−^ mice injected with PBS or AAVDJ‐Lhcgr (8 × 10^9^ gc/testis) 4 weeks after treatment (*n* = 3). The expression levels of marker genes for spermatogonia (*Uchl1*, *Dazl*), spermatocytes (*Sycp3*, *Tex101*), round spermatids (*Acrv1*, *Tssk1*), and elongating spermatids (*Best1*, *Asb9*) were measured in each group. β‐actin was used for normalization. (B–E) Representative images of testicular sections from the three groups 4 weeks after treatment (*n* = 4). Sections were immunostained for PNA (B), DDX4 and TNP2 (D), and counterstained with DAPI. Quantitative analysis showing the percentage of PNA^+^ (C) and TNP2^+^ (E) germ cells in the seminiferous tubules of the testes. Scale bar: 50 μm. Data are expressed as mean ± SEM (A) or boxplots with whiskers showing the minimum to maximum values (C, E). *p < 0.05; **p < 0.01; ***p < 0.001. ns, not significant.

To delineate the molecular consequences of AAVDJ‐Lhcgr on spermatogenesis, we collected testicular tissues from the three groups 4 weeks after AAVDJ‐Lhcgr (8 × 10^9^ gc/testis) treatment. Utilizing qRT‐PCR analysis, we observed that *Lhcgr* ^−/−^ testes in the PBS‐treated group exhibited elevated expression levels of genes associated with spermatogonia (*Uchl1* and *Dazl*) and spermatocytes (*Sycp3* and *Tex101*) (Figure 3A). In contrast, testes treated with AAVDJ‐Lhcgr displayed high expression of genes related to round spermatids (*Acrv1* and *Tssk1*) and elongating spermatids (*Best1* and *Asb9*) (Figure 3A). Immunofluorescence analysis revealed a significant increase in the signal of peanut agglutinin (PNA), which marks the acrosome of spermatids, in the testis of AAVDJ‐Lhcgr‐treated *Lhcgr* ^−/−^ mice (Figure 3B,C). In contrast, the PNA^+^ signals were notably weak in the PBS‐treated group (Figure 3B,C). Moreover, we found discernible expression of the elongating spermatid marker transition protein 2 (TNP2) in the testes of AAVDJ‐Lhcgr‐treated mice, while TNP2 was scarcely detectable in the testes of PBS‐treated *Lhcgr* ^−/−^ mice (Figure 3D,E). Collectively, these findings demonstrate that AAVDJ‐Lhcgr gene therapy significantly promotes the formation of both round and elongating spermatids, indicating its potential to positively impact spermatogenesis in *Lhcgr* ^−/−^ mice.

### 3.5 AAVDJ‐Lhcgr restores fertility and enables the production of fertile offspring by natural mating in *Lhcgr* ^−/−^ mice

**FIGURE 4:**
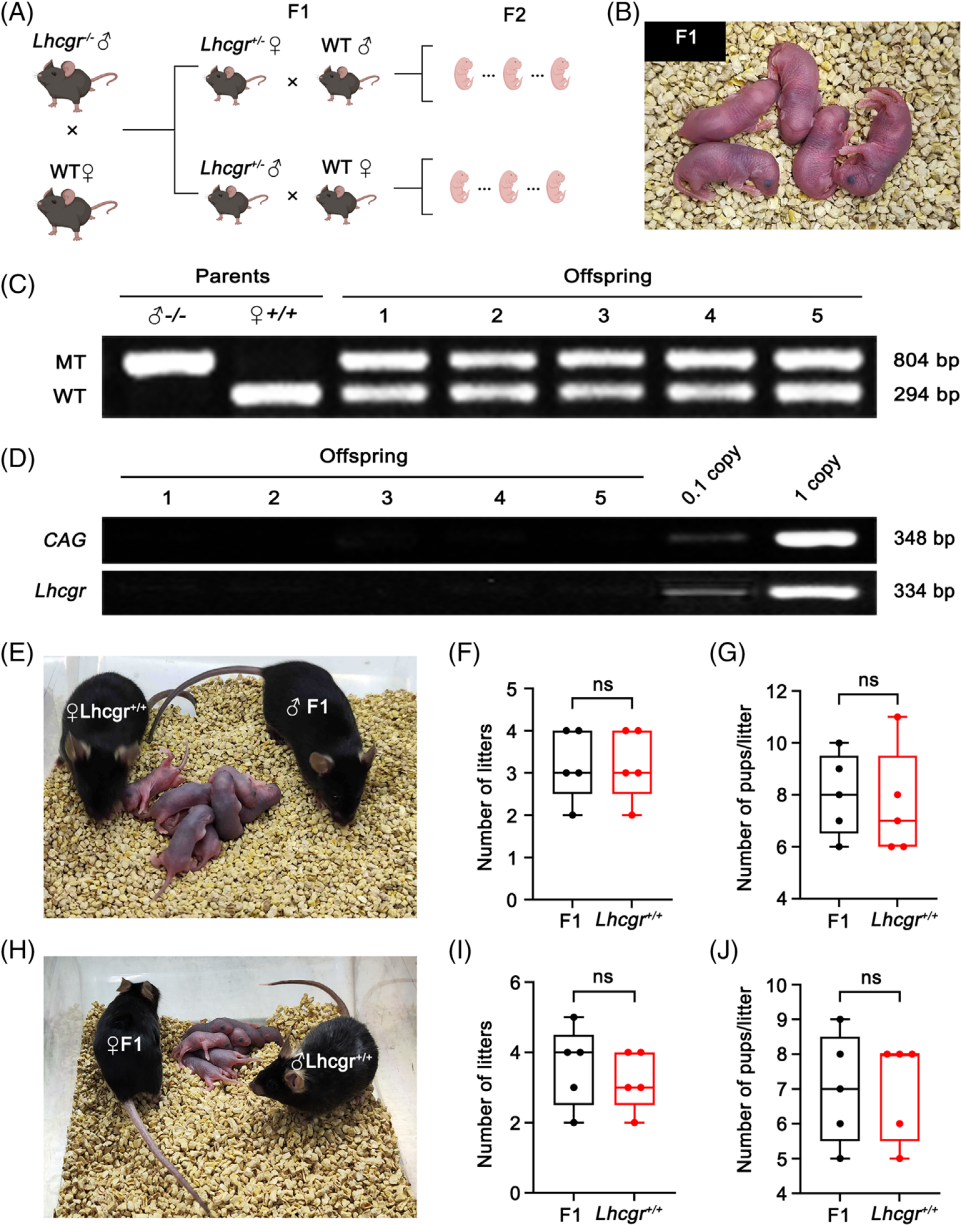
AAVDJ‐Lhcgr restores fertility of *Lhcgr* ^−/−^ male mice and enables production of fertile offspring by natural mating. (A) Mating scheme used to produce the first‐generation (F1) and the second‐generation (F2) mice. (B) Offspring (F1) derived from AAVDJ‐Lhcgr‐treated (8 × 10^9^ gc/testis) *Lhcgr* ^−/−^ male mice. (C) Genotyping of the offspring derived from AAVDJ‐Lhcgr‐treated *Lhcgr* ^−/−^ males and *Lhcgr* ^+/+^ females. The amplified wild‐type (WT, *Lhcgr* ^+/+^) DNA fragment was 294 bp, while the mutant (MT, *Lhcgr* ^−/−^) DNA fragment was 804 bp. (D) PCR analysis of AAVDJ‐Lhcgr integration in the genomes of F1. CAG promoter and Lhcgr‐specific primers were used. As a control, tail DNA from *Lhcgr* ^+/−^ mice was spiked with viral particles representing 0.1 and 1 copies of the viral genome. (E) Male F1 mice were used to produce F2 mice via mating with *Lhcgr* ^+/+^ females. (F and G) Continuous breeding assay starting at 12 weeks of age, showing numbers of litters (F) and pups per litter (G) between F1 males and *Lhcgr* ^+/+^ males within 4 months (*n* = 5). (H) Female F1 mice were used to produce F2 mice via mating with *Lhcgr* ^+/+^ males. (I and J) Continuous breeding assay starting at 12 weeks of age, showing numbers of litters (I) and pups per litter (J) between F1 females and *Lhcgr* ^+/+^ females within 4 months (*n* = 5). Data are represented by boxplots, and whiskers show the minimum to maximum values. ns, not significant.

To assess the fertility of *Lhcgr* ^−/−^ mice following gene therapy, male *Lhcgr* ^−/−^ mice were co‐housed with *Lhcgr* ^+/+^ female mice for breeding 4 weeks after AAVDJ‐Lhcgr (8 × 10^9^ gc/testis) treatment (Figure 4A). After 6 weeks of breeding, 2 out of the 5 *Lhcgr* ^+/+^ female mice gave birth to litters of 5 and 7 offspring, respectively (Figure 4B; Table S2). To validate that the offspring originated from the AAVDJ‐Lhcgr‐treated *Lhcgr* ^−/−^ male mice and *Lhcgr* ^+/+^ females, we performed PCR‐based genotyping on five representative pups. The genotyping analysis confirmed that all five offspring were heterozygous (Figure 4C). Furthermore, to investigate whether AAVDJ integrated into the genomes of the offspring, we extracted DNA from the tails of the offspring born after AAVDJ‐Lhcgr treatment. We then conducted PCR using vector‐specific primers targeting the CAG promoter and exogenous Lhcgr sequence. As a positive control, tail DNA from *Lhcgr* ^+/−^ mice spiked with viral particles was used. Notably, the PCR results did not detect any vector sequence signal for AAVDJ‐Lhcgr in the genome of the detected offspring (Figure 4D; Figure S8), indicating that AAVDJ did not integrate into the genomes of germ cells.

Subsequently, we investigated whether the F1 generated through AAVDJ‐Lhcgr gene therapy could produce the F2. For this purpose, we mated five mature males and five females from the F1 with corresponding *Lhcgr* ^+/+^ mice. Remarkably, all of the F1 mice were capable of producing the F2 through natural mating (Figure 4E,H). Moreover, our observations indicated that the F1 displayed normal fertility comparable to that of *Lhcgr* ^+/+^ mice (Figure 4F,G,I,J). These results further support the favourable outcome of AAVDJ‐Lhcgr treatment in *Lhcgr* ^−/−^ mice, leading to the generation of fertile offspring through natural mating.

### 3.6 AAV‐mediated gene therapy ensures long‐term benefits with a single treatment

**FIGURE 5:**
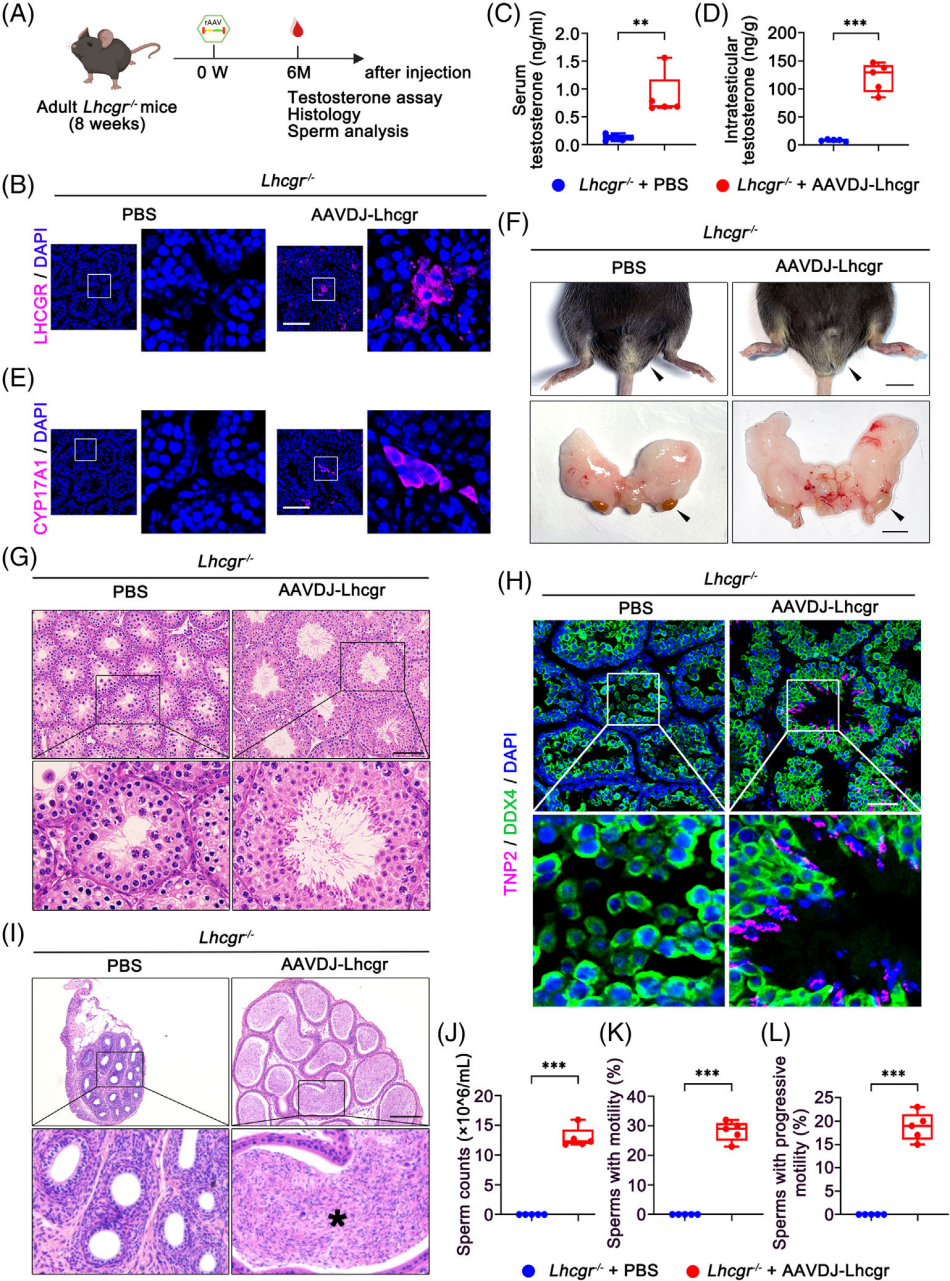
AAV‐mediated gene therapy may ensure long‐term benefits with a single treatment. (A) Experimental overview of the in vivo studies. (B) Representative images of LHCGR expression in the testicular interstitium 6 months after treatment in 8‐week‐old *Lhcgr* ^−/−^ mice injected with PBS or AAVDJ‐Lhcgr (8 × 10^9^ gc/testis) (*n* = 5). The nuclei were counterstained with DAPI. Scale bar: 100 μm. (C and D) The concentrations of serum (C) and intratesticular (D) testosterone were analysed 6 months after treatment in *Lhcgr* ^−/−^ mice injected with PBS or AAVDJ‐Lhcgr (8 × 10^9^ gc/testis) (*n* = 5). (E) Representative images of CYP17A1 expression in the testicular interstitium in *Lhcgr* ^−/−^ mice injected with PBS or AAVDJ‐Lhcgr (8 × 10^9^ gc/testis) 6 months after treatment (*n* = 5). The nuclei were counterstained with DAPI. Scale bar: 100 μm. (F) Representative photographs of the external and internal genitalia of *Lhcgr* ^−/−^ mice injected with PBS or AAVDJ‐Lhcgr (8 × 10^9^ gc/testis) 6 months after treatment (*n* = 5). Arrowheads indicate the testes. Scale bar: 0.5 cm. (G) Representative micrographs of testicular sections from *Lhcgr* ^−/−^ mice injected with PBS or AAVDJ‐Lhcgr (8 × 10^9^ gc/testis) 6 months after treatment (*n* = 5). Arrowhead indicates full spermatogenesis in the testis. Scale bars: 100 μm. (H) Representative images of testicular sections from *Lhcgr* ^−/−^ mice injected with PBS or AAVDJ‐Lhcgr (8 × 10^9^ gc/testis) 6 months after treatment (*n* = 5). Sections were immunostained for DDX4 and TNP2 and counterstained with DAPI. Scale bars: 50 μm. (I) Representative micrographs of cauda epididymal sections from *Lhcgr* ^−/−^ mice injected with PBS or AAVDJ‐Lhcgr (8 × 10^9^ gc/testis) 6 months after treatment (*n* = 5). Stars indicate sperm in the cauda epididymis. Scale bars: 200 μm. (J–L) The sperm counts (J) and proportions of sperm with motility (K) and progressive motility (L) were analysed in *Lhcgr* ^−/−^ mice injected with PBS or AAVDJ‐Lhcgr (8 × 10^9^ gc/testis) 6 months after treatment (*n* = 5). Data are represented by boxplots, and whiskers show the minimum to maximum values. **p < 0.01; ***p < 0.001.

To assess the long‐term therapeutic efficacy of AAVDJ‐Lhcgr, we evaluated functional recovery in *Lhcgr* ^−/−^ mice 6 months after gene therapy (Figure 5A). Immunofluorescence analysis revealed substantial LHCGR expression in the testicular interstitium of the AAVDJ‐Lhcgr (8 × 10^9^ gc/testis) treatment group, while LHCGR was scarcely detectable in *Lhcgr* ^−/−^ mice treated with PBS (Figure 5B). As a result, both serum and intratesticular testosterone concentrations were significantly higher in *Lhcgr* ^−/−^ mice treated with AAVDJ‐Lhcgr compared to those in PBS‐treated *Lhcgr* ^−/−^ mice (Figure 5C,D). Furthermore, immunofluorescence analysis demonstrated an increased number of CYP17A1^+^ LCs in the testes of the AAVDJ‐Lhcgr‐treated group when compared to the PBS‐treated mice (Figure 5E). Notably, the masculinization characteristics observed in *Lhcgr* ^−/−^ mice treated with AAVDJ‐Lhcgr for 6 months remained evident, in contrast to PBS‐treated *Lhcgr* ^−/−^ mice (Figure 5F).

Additionally, histological analysis revealed remarkable improvement in spermatogenesis in AAVDJ‐Lhcgr‐treated *Lhcgr* ^−/−^ mice, as evidenced by the presence of spermatozoa within the seminiferous tubules (Figure 5G). Immunofluorescence staining demonstrated a significant increase in the number of TNP2^+^ cells in *Lhcgr* ^−/−^ testes after 6 months of AAVDJ‐Lhcgr treatment compared to PBS‐treated testes (Figure 5H). Moreover, histological staining of the cauda epididymis in *Lhcgr* ^−/−^ mice revealed massive sperm following AAVDJ‐Lhcgr treatment (Figure 5I). We further quantified sperm count and motility using the CASA system. The results demonstrated a persistent and significant improvement in sperm count and motility in AAVDJ‐Lhcgr‐treated *Lhcgr* ^−/−^ mice after 6 months of gene therapy, while no sperm were detected in PBS‐treated *Lhcgr* ^−/−^ mice (Figure 5J–L). Collectively, these findings provide compelling evidence that AAVDJ‐Lhcgr treatment leads to enduring and beneficial effects with a single treatment in *Lhcgr* ^−/−^ mice.

### 3.7 AAVDJ‐Lhcgr improves adipose and muscle function and promotes bone formation in *Lhcgr* ^−/−^ mice

**FIGURE 6:**
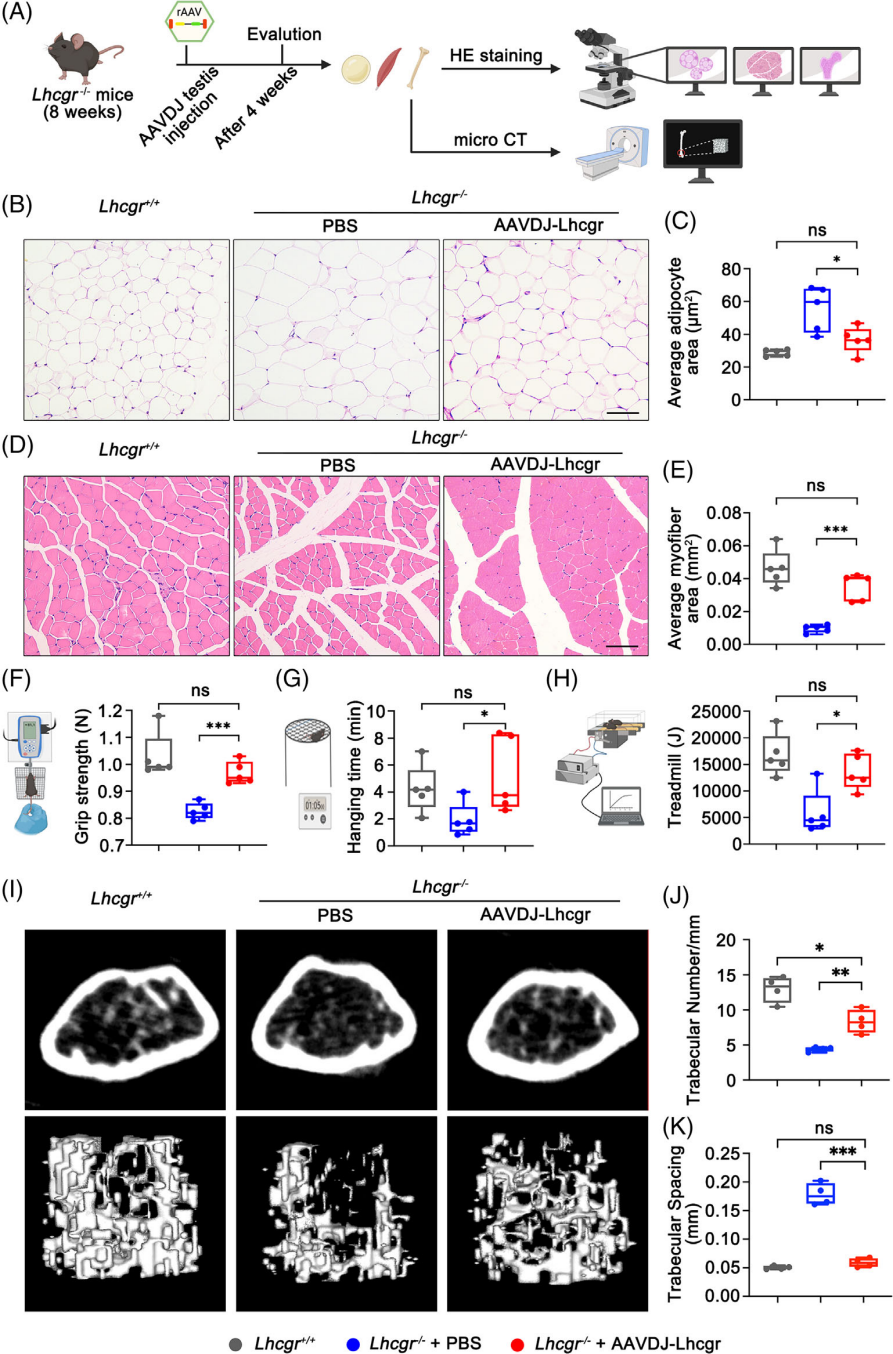
AAVDJ‐Lhcgr improves adipose and muscle function and promotes bone formation in *Lhcgr* ^−/−^ mice. (A) Experimental overview of the study to assess adipose tissue, muscle and bone. (B and C) Representative images of H&E staining (B) and quantification of the average adipocyte area (C) of epididymal fat isolated from *Lhcgr* ^+/+^ mice and *Lhcgr* ^−/−^ mice injected with PBS or AAVDJ‐Lhcgr (8 × 10^9^ gc/testis) 4 weeks after treatment (*n* = 5). Scale bar: 200 μm. (D and E) Representative images of H&E staining (D) and quantification of the average myofiber area (E) of tibialis anterior muscle isolated from *Lhcgr* ^+/+^ mice and *Lhcgr* ^−/−^ mice injected with PBS or AAVDJ‐Lhcgr (8 × 10^9^ gc/testis) 4 weeks after treatment (*n* = 5). Scale bar: 200 μm. (F–H) Assessment of grip strength (F), hanging time (G) and treadmill (H) of *Lhcgr* ^+/+^ mice and *Lhcgr* ^−/−^ mice injected with PBS or AAVDJ‐Lhcgr (8 × 10^9^ gc/testis) 4 weeks after treatment (*n* = 5). (I–K) Representative micro‐CT images (I) and quantification of the trabecular number (J), and trabecular spacing (K) of distal femurs isolated from *Lhcgr* ^+/+^ mice and *Lhcgr* ^−/−^ mice injected with PBS or AAVDJ‐Lhcgr (8 × 10^9^ gc/testis) 4 weeks after treatment (*n* = 4). Data are represented by boxplots, and whiskers show the minimum to maximum values. *p < 0.05; **p < 0.01; ***p < 0.001. ns, not significant.

Previous studies have established a connection between low testosterone levels and adverse effects on body composition, including increased body fat, reduced muscle mass, and impaired muscle function.^7^ In our current investigation, we examined the impact of AAVDJ‐Lhcgr treatment on these parameters in *Lhcgr* ^−/−^ mice (Figure 6A). After 4 weeks of AAVDJ‐Lhcgr (8 × 10^9^ gc/testis) treatment, we observed a significant decrease in the epididymal fat‐to‐body weight ratio in AAVDJ‐Lhcgr‐treated *Lhcgr* ^−/−^ mice compared to those treated with PBS (Figure S9A). Histological analysis further revealed that the adipocytes of epididymal fat were smaller in size in the AAVDJ‐Lhcgr‐treated group, in stark contrast to the significantly larger adipocyte size observed in the PBS‐treated group (Figure 6B,C). Furthermore, the weight of the tibialis anterior muscle in *Lhcgr* ^−/−^ mice treated with AAVDJ‐Lhcgr was significantly greater than that of mice in the PBS‐treated group (Figure S9B). Histological examination of the tibialis anterior muscle indicated a substantial increase in cross‐sectional myofiber area in *Lhcgr* ^−/−^ mice after AAVDJ‐Lhcgr treatment compared to the PBS‐treated group (Figure 6D,E). In addition, we conducted grip strength tests and found that *Lhcgr* ^−/−^ mice treated with AAVDJ‐Lhcgr exhibited a significantly greater grip strength compared to mice in the PBS‐treated group 4 weeks after treatment (Figure 6F). The hanging time was notably increased in *Lhcgr* ^−/−^ mice treated with AAVDJ‐Lhcgr, indicating enhanced muscle endurance after gene therapy (Figure 6G). Moreover, treadmill tests revealed a marked improvement in exercise capacity in *Lhcgr* ^−/−^ mice subjected to AAVDJ‐Lhcgr treatment compared to PBS‐treated mice (Figure 6H). Overall, these findings demonstrate the beneficial effects of AAVDJ‐Lhcgr treatment on body fat reduction, muscle mass enhancement and muscle function improvement in *Lhcgr* ^−/−^ mice.

Previous studies have established a link between testosterone deficiency and a reduction in bone mineral density.^7^ Therefore, we next examined the impact of AAVDJ‐Lhcgr treatment on bone parameters in *Lhcgr* ^−/−^ mice. The femur weight of *Lhcgr* ^−/−^ mice treated with AAVDJ‐Lhcgr exhibited a significant increase compared to those of mice in the PBS‐treatment group (Figure S9C). Morphological assessment of femur tissue from all three groups revealed a noticeable decrease in the number of adipocytes in the bone marrow cavity of *Lhcgr* ^−/−^ mice following AAVDJ treatment (Figure S9D,E), indicating the inhibitory effect of gene therapy on fat accumulation in bone marrow. To further investigate bone microarchitecture, we conducted micro‐CT scans on the right femur of the three groups. We analysed micro‐CT scan images of the horizontal and coronal surfaces of the femur and revealed higher trabecular bone number and thickness but lower trabecular bone spacing in AAVDJ‐Lhcgr‐treated *Lhcgr* ^−/−^ mice compared to those in mice treated with PBS (Figure 6I–K; Figure S9F). Collectively, these results suggest that AAVDJ‐based gene therapy holds promise in safeguarding skeletal homeostasis by curbing marrow fat accumulation and promoting bone formation in *Lhcgr* ^−/−^ mice.

## 4 DISCUSSION

AAVDJ is a variant generated from the libraries of AAV hybrids of eight serotypes by DNA shuffling method.^26^ Previous studies have reported that AAVDJ is able to deliver higher quantities of therapeutic DNA both in vitro and in vivo.^26^, ^27^ However, the use of AAVDJ in testis has not been previously reported. Here, in order to identify AAV vectors which can more efficiently target Leydig cell progenitors and ensure more robust gene expression, we screened a panel of AAV subtypes and observed that AAVDJ achieved markedly efficient transduction of Leydig cell progenitors in *Lhcgr* ^−/−^ mice. While there is a concern that high concentrations of AAV vectors might induce heightened immune responses leading to potential transduced cell clearance and decreased therapy efficiency,^28^ our analysis did not find any significant difference in CD4^+^ and CD8^+^ lymphocyte infiltration post‐AAVDJ administration at the highest dose (8 × 10^10^ gc/testis) when compared to uninjected controls after 7 days. This observation highlights the potential safety and tolerance of interstitial AAVDJ injections. A critical factor limiting the broader application of gene therapy to patients who could benefit is the high cost associated with AAV production.^29^ In the present study, AAVDJ significantly enhanced the functionality of LCs and supported reproductive organ development in *Lhcgr* ^−/−^ mice, even with a 10‐fold reduction in genome copy numbers compared to the high‐dose AAV8 administration.^12^ In light of our findings that reduce the AAV dosage required for a favourable therapeutic outcome, there exists a notable opportunity to mitigate associated financial burdens. Subsequently, this paves the way for extending the therapeutic advantages of gene therapy to a larger subset of patients diagnosed with LCF.

Restoration of fertility is an urgent need for LCF patients.^30^ Conventional established therapies have demonstrated limited success in addressing this critical issue.^9^ Our recent work has shown promising outcomes using AAV8‐mediated gene therapy to rescue spermatogenesis in *Lhcgr* ^−/−^ mice.^12^ However, the limited recovery of sperm counts and testosterone levels in AAV8‐Lhcgr treated *Lhcgr* ^−/−^ mice hindered successful production of the F1 by natural mating. As a result, it became necessary to generate offspring through IVF in our previous study.^12^ In contrast, AAVDJ gene therapy achieved significant improvements in the testosterone levels in *Lhcgr* ^−/−^ mice, reaching approximately 60% of the testosterone level observed in *Lhcgr* ^+/+^ mice. Notably, the sperm count and motility were almost completely restored in AAVDJ‐Lhcgr‐treated *Lhcgr* ^−/−^ mice. Due to these remarkable enhancements in testosterone production and sperm parameters, the treated LCF mice were able to naturally sire offspring with *Lhcgr* ^+/+^ female mice. Moreover, the F1 mice were able to produce the F2 through natural breeding with *Lhcgr* ^+/+^ mice and showed normal fertility. Previous studies have raised concerns about the potential integration of AAV sequences into the cell genome,^31^ although most evidence suggests that AAV lacks this capability.^11^ In the current study, we conducted PCR analysis to examine the genomic DNA from the F1, and no AAV‐related sequences were detected. Such an observation further strengthens the evidence supporting the reproductive safety of AAVDJ‐mediated gene therapy in the treatment of LCF. Our findings are in line with previous research on AAV1 and AAV8, which also did not show integration into the germ cell genome following injection into mouse testes.^12^, ^21^ These findings collectively highlight the potential of AAVDJ‐mediated gene therapy for LCF treatment to address the urgent need for restoration of fertility in affected individuals.

Previous researches have consistently reported that patients with low testosterone level experience increased adiposity,^13^ reduced skeletal muscle mass^14^ and decreased bone mineral density,^15^ and TRT has been shown to mitigate these issues.^8^ In the present study, AAVDJ‐mediated gene therapy ameliorated adipose tissue accumulation, enhanced skeletal muscle mass, strengthened muscle functionality and preserved skeletal homeostasis in *Lhcgr* ^−/−^ mice. These favourable results imply that AAVDJ‐mediated gene therapy can improve physical function impaired by testosterone deficiency. Furthermore, serum testosterone levels remained elevated in *Lhcgr* ^−/−^ mice treated with AAVDJ even after 6 months, indicating the potential for long‐term benefits from a single AAVDJ treatment. As reported, testosterone deficiency affects a considerable portion of men aged 40–70 years, leading to increased fat mass, decreased muscle mass, osteoporosis and metabolic syndrome.^7^, ^32^ Given these favourable improvements and long‐term testosterone recovery in the current study, we anticipate that AAVDJ‐mediated gene therapy may serve as a promising strategy for addressing aging‐related testosterone deficiency. Specific experiments to evaluate its feasibility and efficacy for this purpose are urgently needed in future studies.

In conclusion, our study demonstrated the effectiveness and safety of AAVDJ‐mediated gene therapy as a promising treatment for LCF. Even at a relatively low dose, AAVDJ gene therapy significantly increased serum testosterone levels, substantially improved sexual development, and partially promoted spermatogenesis in *Lhcgr* ^−/−^ mice. Importantly, AAVDJ‐mediated gene therapy enabled the restoration of fertility in *Lhcgr* ^−/−^ mice through natural mating, leading to the birth of the second‐generation offspring. Additionally, this therapeutic approach effectively improved the function of adipose, muscle and bone in *Lhcgr* ^−/−^ mice. These findings provide compelling evidence for the potential clinical applications of gene therapies targeting genetic LCF or other reproductive diseases.

## AUTHOR CONTRIBUTIONS

S.Z., B.Y., X.S. and H.C. contributed equally to this work. S.Z. and B.Y. carried out the experiments, assisted with the experimental design and wrote the manuscript. X.S. assisted with the design of the experiments and data analysis. H.C. carried out the experiments and data analysis. F.W. assisted with the experimental design. Z.T. assisted with the design of the viral vector. W.O., C.Y. and C.L. assisted with animal experiments. H.P. and P.L. assisted with data analysis. L.P., Z.L. and S.Y. assisted with manuscript revision. T.W., Q.K. and C.D. assisted with the experimental design and revised the manuscript. A.P.X. and K.X. conceived the project, supervised all experiments, and wrote and revised the manuscript. All authors fulfil the criteria for authorship.

## FUNDING INFORMATION

This study was supported by National Key Research and Development Program of China, Grant/Award Number: 2022YFA1104100; National Natural Science Foundation of China, Grant/Award Number: 32130046, 82371611, 82371609, 82171564, 82101669; Key Research and Development Program of Guangdong Province, Grant/Award Number: 2019B020235002; Natural Science Foundation of Guangdong Province, Grant/Award Number: 2022A1515010371; Guangdong Basic and Applied Basic Research Foundation, Grant/Award Number: 2021A1515010377; Key Scientific and Technological Program of Guangzhou City, Grant/Award Number: 2023B01J1002; Pioneering talents project of Guangzhou Development Zone, Grant/Award Number: 2021‐L029.

## CONFLICT OF INTEREST STATEMENT

The authors declare no conflicts of interest.

## Supporting information


**Figure S1:** AAVDJ shows the highest transfection efficiency to testicular cells.
**Figure S2:** Testicular injection of AAVDJ targets Leydig cell progenitors.
**Figure S3:** AAVDJ shows testis tropism after intratesticular injection.
**Figure S4:** Inflammatory cells infiltration after AAVDJ‐mCherry injection.
**Figure S5:** Characteristics of serum testosterone levels after degarelix or hCG injection.
**Figure S6:** AAVDJ‐Lhcgr treatment promotes proliferation and differentiation of Leydig cell progenitors.
**Figure S7:** AAVDJ‐Lhcgr promotes reproductive organ development in Lhcgr^−/−^ mice.
**Figure S8:** PCR analysis of AAVDJ‐Lhcgr integration in the genomes of F1.
**Figure S9:** AAVDJ‐Lhcgr improves physical function in Lhcgr^−/−^ mice.

**Table S1:** Primers used to amplify the transcripts in PCR analysis.
**Table S2:** Progeny from *Lhcgr* ^*−/−*^ mice injected with AAVDJ‐Lhcgr.

## Data Availability

The data that support the findings of this study are available from the corresponding author upon reasonable request.
