# Supplementary material for: AAV‐mediated gene therapy restores natural fertility and improves physical function in the Lhcgr‐deficient mouse model of Leydig cell failure
Source: Cell Prolif. 2024 May 30;57(9):e13680. doi: 10.1111/cpr.13680 (PMC11503244; doi:10.1111/cpr.13680)
Supplement: Supplementary file 1 — Figure S1: AAVDJ shows the highest transfection efficiency to testicular cells. Figure S2: Testicular injection of AAVDJ targets Leydig cell progenitors. Figure S3: AAVDJ shows testis tropism after intratesticular injection. Figure S4: Inflammatory cells infiltration after AAVDJ‐mCherry injection. Figure S5: Characteristics of serum testosterone levels after degarelix or hCG injection. Figure S6: AAVDJ‐Lhcgr treatment promotes proliferation and differentiation of Leydig cell progenitors. Figure S7: AAVDJ‐Lhcgr promotes reproductive organ development in Lhcgr−/− mice. Figure S8: PCR analysis of AAVDJ‐Lhcgr integration in the genomes of F1. Figure S9: AAVDJ‐Lhcgr improves physical function in Lhcgr−/− mice. [file CPR-57-e13680-s002.pdf]

# SUPPORTING INFORMATION

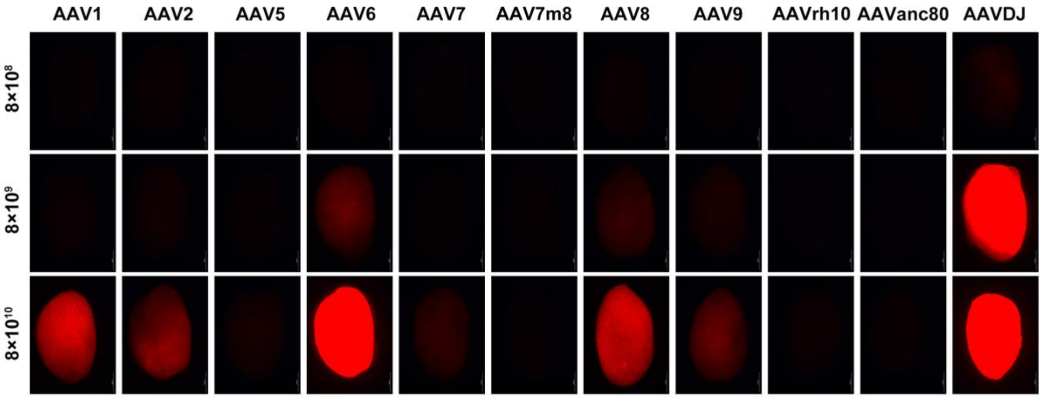


**Figure S1. AAVDJ shows the highest transfection efficiency to testicular cells.**

Macroscopic appearance of wild-type mouse testes 7 days after microinjection with mCherry-expressing AAVs with different capsids (1, 2, 5, 6, 7, 7m8, 8, 9, rh10, anc80, and DJ) at increasing doses of $8\times10^{8}$, $8\times10^{9}$, and $8\times10^{10}$ gc/testis, respectively (n=3).

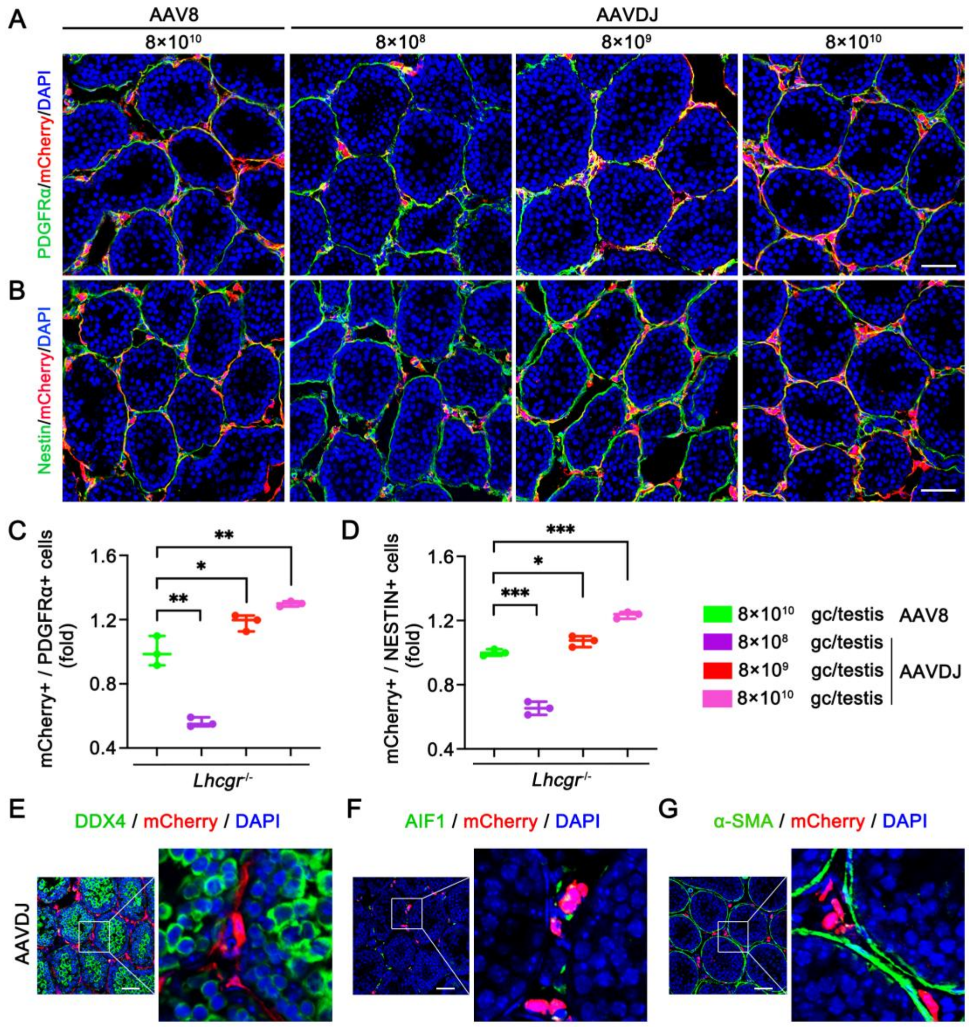


**Figure S2. Testicular injection of AAVDJ targets Leydig cell progenitors.**

(A and B) Representative confocal images of the testicular sections of *Lhcgr*$^{-/-}$ mice injected with increasing doses of AAVDJ-mCherry ($8×10^8$, $8×10^9$, and $8×10^{10}$ gc/testis) or AAV8-mCherry ($8×10^{10}$ gc/testis). The testicular tissues were collected and immunostained with Leydig cell progenitors markers (PDGFRα, Nestin) 7 days after AAV injection (n=3). Scale bars: 50 μm. (C and D) Viral transduction rates were determined from the number of mCherry$^+$ cells divided by the number of PDGFRα$^+$ or Nestin$^+$ Leydig cell progenitors. Data are represented by plots, and whiskers are minimum to maximum values. *p < 0.05, **p < 0.01, ***p < 0.001. (E-G)

Immunostaining of AAVDJ-mCherry-injected ($8\times10^{10}$ gc/testis) testes for the germ cell marker DDX4 (E), macrophage markers AIF1 (F), and peritubular myoid cell marker α-SMA (G) 7 days after injection (n=3). Nuclei were counterstained with DAPI. Scale bar: 50 μm.

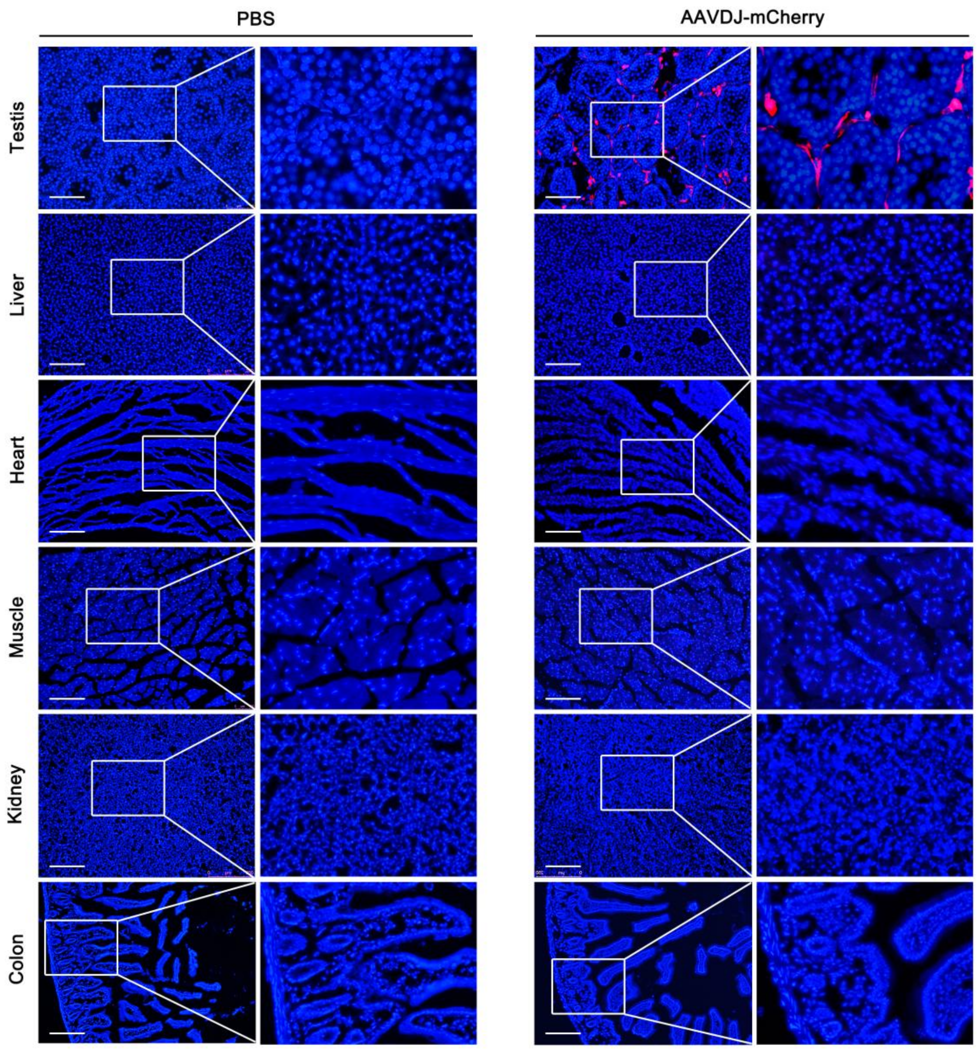


**Figure S3. AAVDJ shows testis tropism after intratesticular injection.**

Representative fluorescent photographs of mCherry expression (red) in indicated organs of AAVDJ-mCherry ($8\times10^{10}$ gc/testis) treated mice 4 weeks after intratesticular injection. Scale bar: 200 μm.

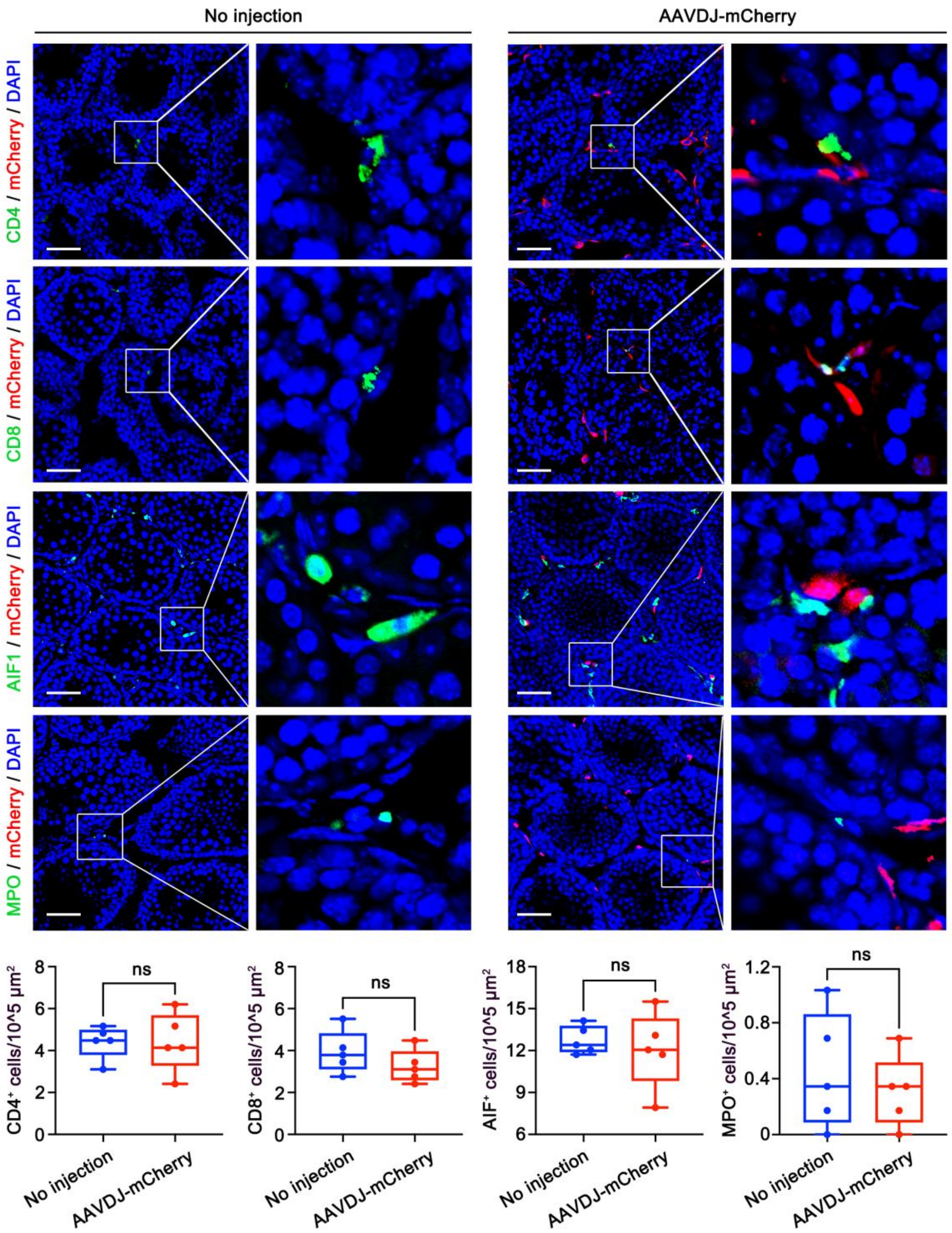


**Figure S4. Inflammatory cells infiltration after AAVDJ-mCherry injection.**

Immunostaining and quantitative analysis with CD4, CD8, AIF1 and MPO antibodies in *Lhcgr*$^{-/-}$ mouse testes 7 days after microinjection AAVDJ-mCherry ($8\times10^{10}$ gc/testis; n=5) . Nuclei were counterstained with DAPI. Scale bar: 50 μm. Data are represented by boxplots, and whiskers show the minimum to maximum values. ns=not significant.

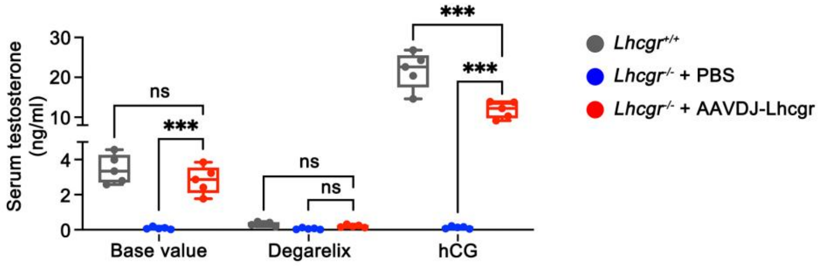


**Figure S5. Characteristics of serum testosterone levels after degarelix or hCG injection.**

The concentrations of serum testosterone were analyzed before and after degarelix or hCG injection in *Lhcgr*$^{+/+}$ mice, and *Lhcgr*$^{-/-}$ mice injected with PBS or AAVDJ-Lhcgr (n=5). Data are represented by boxplots, and whiskers show the minimum to maximum values. ***$p < 0.001$, ns=not significant.

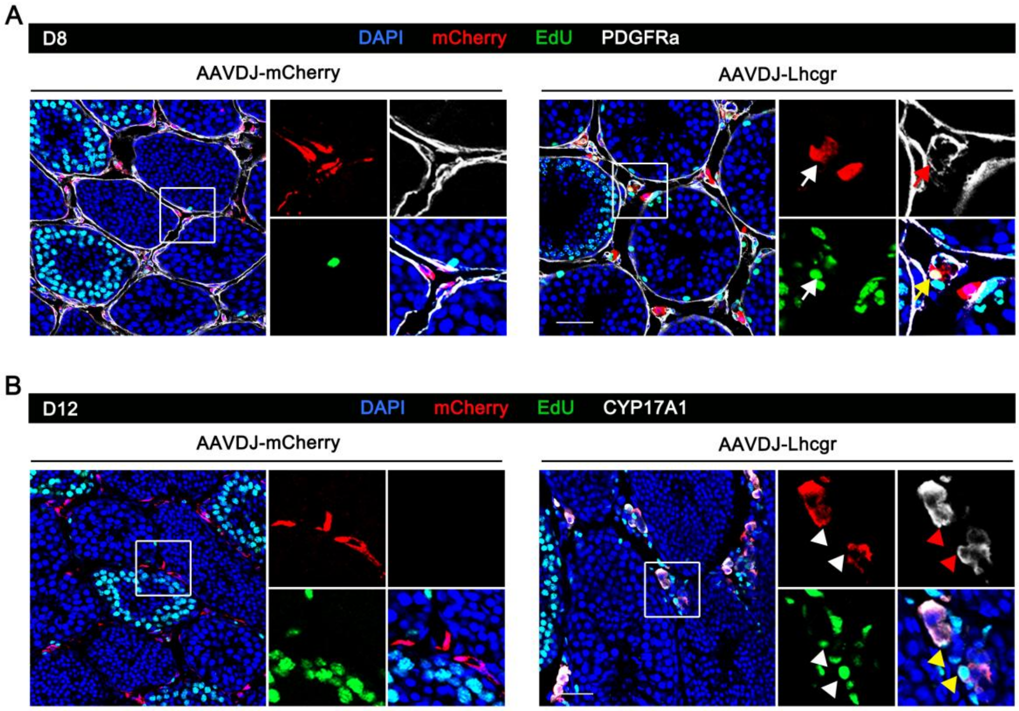


**Figure S6. AAVDJ-Lhcgr treatment promotes proliferation and differentiation of Leydig cell progenitors.**

(A) Representative confocal images of EdU, mCherry, and PDGFRα in the testicular sections from *Lhcgr*$^{-/-}$ mice injected AAVDJ-mCherry ($8\times10^9$ gc/testis) or AAVDJ-Lhcgr ($8\times10^9$ gc/testis) at day 8 (n=3). Arrows indicate the proliferation of Leydig cell progenitors. Nuclei were counterstained with DAPI. Scale bar: 50 μm. (B) Representative confocal images of EdU, mCherry, and CYP17A1 in the testicular sections from *Lhcgr*$^{-/-}$ mice injected AAVDJ-mCherry ($8\times10^9$ gc/testis) or AAVDJ-Lhcgr ($8\times10^9$ gc/testis) at day 12 (n=3). Arrowheads indicate the differentiation of Leydig cell progenitors. Nuclei were counterstained with DAPI. Scale bar: 50 μm.

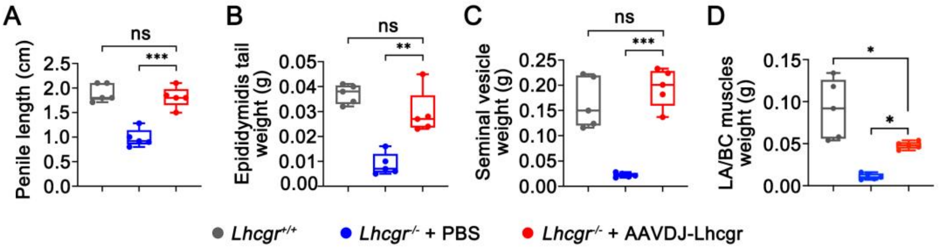


**Figure S7. AAVDJ-Lhcgr promotes reproductive organ development in *Lhcgr*$^{-/-}$ mice.**

(A-D) Quantification of penile length (A), epididymis tail weight (B), seminal vesicle weight (C), and LA/BC (D) of *Lhcgr*$^{+/+}$ mice and *Lhcgr*$^{-/-}$ mice injected with PBS or AAVDJ-Lhcgr ($8\times10^9$ gc/testis) 4 weeks after treatment (n=5). LA/BC, levator ani/bulbocavernosus weight. Data are represented by boxplots, and whiskers show the minimum to maximum values. *$p < 0.05$, **$p < 0.01$, ***$p < 0.001$, ns=not significant.

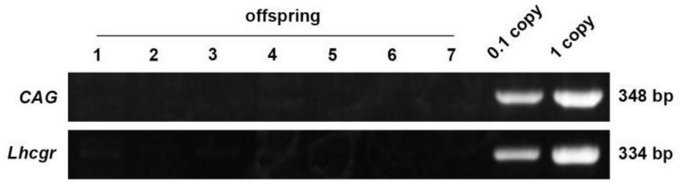


**Figure S8. PCR analysis of AAVDJ-Lhcgr integration in the genomes of F1.**

CAG promoter and Lhcgr-specific primers were used. As a control, tail DNA from *Lhcgr*$^{+/-}$ mice was spiked with viral particles representing 0.1 and 1 copies of the viral genome.

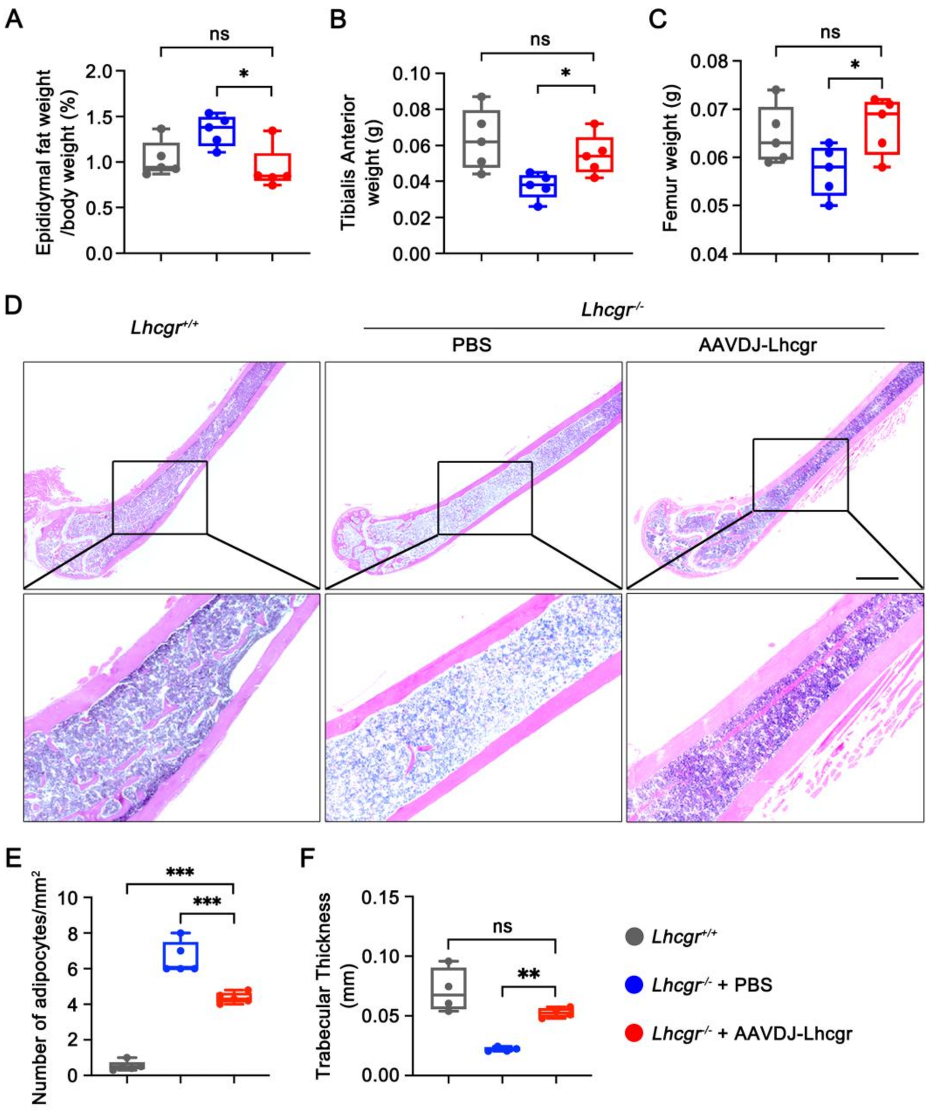


**Figure S9. AAVDJ-Lhcgr improves physical function in *Lhcgr*$^{-/-}$ mice.**

(A-C) Quantification of epididymal fat weight/body weight (A), weight of tibialis anterior muscle (B), and weight of femur (C) in *Lhcgr*$^{+/+}$ mice and *Lhcgr*$^{-/-}$ mice injected with PBS or AAVDJ-Lhcgr (8×10$^{9}$ gc/testis) 4 weeks after treatment (n=5). (D and E) Representative images of H&E staining (D) and quantification of adipocyte number (E) of distal femurs obtained from *Lhcgr*$^{+/+}$ mice and *Lhcgr*$^{-/-}$ mice injected with PBS or AAVDJ-Lhcgr (8×10$^{9}$ gc/testis) 4 weeks after treatment (n=5). Scale bar: 1 mm. (F) Quantification of trabecular thickness in *Lhcgr*$^{+/+}$ mice and *Lhcgr*$^{-/-}$ mice

injected with PBS or AAVDJ-Lhcgr ($8\times10^9$ gc/testis) 4 weeks after treatment (n=4). Data are represented by boxplots, and whiskers show the minimum to maximum values. *$p < 0.05$, **$p < 0.01$, ***$p < 0.001$, ns=not significant.
