# Supplementary material for: AAV‐mediated gene therapy restores natural fertility and improves physical function in the Lhcgr‐deficient mouse model of Leydig cell failure
Source: Cell Prolif. 2024 May 30;57(9):e13680. doi: 10.1111/cpr.13680 (PMC11503244; doi:10.1111/cpr.13680)
Supplement: Supplementary file 2 — Table S1: Primers used to amplify the transcripts in PCR analysis. Table S2: Progeny from Lhcgr −/− mice injected with AAVDJ‐Lhcgr. [file CPR-57-e13680-s001.docx]

**Table S1. Primers used to amplify the transcripts in PCR analysis.**

| **Primers for qRT-PCR** | | | |
| --- | --- | --- | --- |
| **Gene** | **Forward Primer** | **Reverse Primer** | |
| *Lhcgr* | CACTCTCCAGAGTTGTCAGGG | GAGGTTTGTAAAAGCACCGGG | |
| *Dazl* | CTTCATCAGCAACCACAA | TTCATCCATCCTAACATCAAT | |
| *Uchl* | TGGAATTTGAGGATGGAT | AACACTTGGCTCTATCTT | |
| *Sycp3* | CGCTGAGCAAACATCTAAAGA | CAACCAAAGGTGGCTTCC | |
| *Tex101* | TACCTTTAACTGGACTTCA | CCATCTGCTTTAATCAACA | |
| *Acrv1* | CAGGTGAACAGGTGTCTA | CAGATGTGCTTGGAAGTG | |
| *Tssk1* | CAAGGACTTCAACATCAA | GGTCTTGCTTAATATCAGT | |
| *Best1* | AACTTGAACATTCCAGAG | TCATTAGAGCCTGTATATTG | |
| *Asb9* | ACTATAACATCAGCCATC | CCTTGATTCACAGATACT | |
| **Primers for Integration Assay** | | | |
| *CAG* | TTCGGCTTCTGGCGTGTGA | | GGTGAGAGATAGTCGGGCG |
| *Lhcgr* | AGCTAATGCCTTTGACAACCTC | | CGAGATTAGCGTCGTCCCAT |

**Table S2. Progeny from *Lhcgr^-/-^* mice injected with AAVDJ-Lhcgr.**

| **Exp** | **No. of animals** | **Fertile animals (%)** | **Virus titer** | **Days to progeny** | **Number of offspring** |
| --- | --- | --- | --- | --- | --- |
| No. 1 | 3  2 | 1 (33.3%) | 8×10^9^ | 30 | 5 |
| No. 2 |  | 1 (50%) | 8×10^9^ | 34 | 7 |

All AAVDJ-Lhcgr treated *Lhcgr^-/-^* male mice were mated with *Lhcgr*^+/+^ female mice to examine the fertility after gene therapy.
